# Systematic decoding of *cis* gene regulation defines context-dependent control of the multi-gene costimulatory receptor locus in human T cells

**DOI:** 10.1038/s41588-024-01743-5

**Published:** 2024-05-29

**Authors:** Cody T. Mowery, Jacob W. Freimer, Zeyu Chen, Salvador Casaní-Galdón, Jennifer M. Umhoefer, Maya M. Arce, Ketrin Gjoni, Bence Daniel, Katalin Sandor, Benjamin G. Gowen, Vinh Nguyen, Dimitre R. Simeonov, Christian M. Garrido, Gemma L. Curie, Ralf Schmidt, Zachary Steinhart, Ansuman T. Satpathy, Katherine S. Pollard, Jacob E. Corn, Bradley E. Bernstein, Chun Jimmie Ye, Alexander Marson

**Affiliations:** 1 Medical Scientist Training Program, University of California, San Francisco, San Francisco, CA USA; 2 Biomedical Sciences Graduate Program, University of California, San Francisco, San Francisco, CA USA; 3 Gladstone-UCSF Institute of Genomic Immunology, San Francisco, CA USA; 4 Department of Medicine, University of California, San Francisco, San Francisco, CA USA; 5 Department of Microbiology and Immunology, University of California, San Francisco, San Francisco, CA USA; 6 Department of Genetics, Stanford University, Stanford, CA USA; 7 Department of Cancer Biology, Dana–Farber Cancer Institute, Boston, MA USA; 8 Broad Institute of MIT and Harvard, Cambridge, MA USA; 9 Departments of Cell Biology and Pathology, Harvard Medical School, Boston, MA USA; 10 Gladstone Institute of Data Science and Biotechnology, San Francisco, CA USA; 11 Department of Epidemiology and Biostatistics, University of California, San Francisco, San Francisco, CA USA; 12 Department of Pathology, Stanford University, Stanford, CA USA; 13 Center for Personal Dynamic Regulomes, Stanford University, Stanford, CA USA; 14 Innovative Genomics Institute, University of California, Berkeley, Berkeley, CA USA; 15 Department of Molecular and Cell Biology, University of California, Berkeley, Berkeley, CA USA; 16 Diabetes Center, University of California, San Francisco, San Francisco, CA USA; 17 Department of Surgery, University of California, San Francisco, San Francisco, CA USA; 18 UCSF CoLabs, University of California, San Francisco, San Francisco, CA USA; 19 Parker Institute for Cancer Immunotherapy, San Francisco, CA USA; 20 Program in Immunology, Stanford University, Stanford, CA USA; 21 Chan Zuckerberg Biohub SF, San Francisco, CA USA; 22 Rosalind Russell/Ephraim P. Engleman Rheumatology Research Center, University of California, San Francisco, San Francisco, CA USA; 23 Bakar Computational Health Sciences Institute, University of California, San Francisco, San Francisco, CA USA; 24 Institute for Human Genetics, University of California, San Francisco, San Francisco, CA USA; 25 UCSF Helen Diller Family Comprehensive Cancer Center, University of California, San Francisco, San Francisco, CA USA; 26 Present Address: Department of Microchemistry, Proteomics, Lipidomics and Next Generation Sequencing, Genentech, South San Francisco, CA USA; 27 Present Address: Department of Laboratory Medicine, Medical University of Vienna, Vienna, Austria; 28 Present Address: Department of Biology, ETH Zürich, Zürich, Switzerland

**Keywords:** Immunogenetics, Gene regulation

## Abstract

*Cis*-regulatory elements (CREs) interact with *trans* regulators to orchestrate gene expression, but how transcriptional regulation is coordinated in multi-gene loci has not been experimentally defined. We sought to characterize the CREs controlling dynamic expression of the adjacent costimulatory genes *CD28*, *CTLA4* and *ICOS*, encoding regulators of T cell-mediated immunity. Tiling CRISPR interference (CRISPRi) screens in primary human T cells, both conventional and regulatory subsets, uncovered gene-, cell subset- and stimulation-specific CREs. Integration with CRISPR knockout screens and assay for transposase-accessible chromatin with sequencing (ATAC-seq) profiling identified *trans* regulators influencing chromatin states at specific CRISPRi-responsive elements to control costimulatory gene expression. We then discovered a critical CCCTC-binding factor (CTCF) boundary that reinforces CRE interaction with *CTLA4* while also preventing promiscuous activation of *CD28*. By systematically mapping CREs and associated *trans* regulators directly in primary human T cell subsets, this work overcomes longstanding experimental limitations to decode context-dependent gene regulatory programs in a complex, multi-gene locus critical to immune homeostasis.

## Abstract (web summary)

Functional characterization of the regulatory landscape of the adjacent costimulatory genes *CD28*, *CTLA4* and *ICOS* in primary human T cell subsets identifies context-dependent programs controlling this locus critical for immune homeostasis.

## Main

Interactions of CREs and *trans* regulators control how genes are expressed in specific cell types and in response to specific extracellular stimuli^1,2^. Context-restricted transcription factors work in concert with chromatin-modifying complexes to bind CREs and tune the expression of target transcriptional programs^3–6^. However, how CREs and *trans* regulators coordinate to control gene expression in complex multi-gene loci harboring one or more functionally related genes remains unknown^7^. While adjacent genes are commonly transcriptionally coexpressed^8–11^, many loci harbor multiple genes that exhibit divergent expression patterns. By organizing the genome into topologically associating domains (TADs) and subTADs, regulators of chromatin structure such as CTCF play critical roles orchestrating transcriptional regulation by promoting interactions between CREs and target gene promoters^12–15^ while insulating nontarget loci from promiscuous activation^16–20^. Natural genetic variation in CREs can disrupt these modes of transcriptional regulation and confer risk for disease^21^, providing strong motivation to functionally decode CREs and *trans* regulators directly in disease-relevant primary human cells.

We sought to map systematically the CREs influencing expression of three critical immune genes: *CD28*, *CTLA4* and *ICOS*. These ‘costimulatory genes’ are encoded adjacently on human chromosome (chr) 2q33.2 and likely arose from ancestral duplications of *CD28* (refs. ^22,23^). With evolution, each gene functionally diverged^24^ and acquired distinct expression dynamics^25^. The genes encode surface receptors that influence the functional outcome of T cell activation and, thus, regulate immune homeostasis more broadly^26^. *CD28* is constitutively expressed, and engagement of the cluster of differentiation 28 (CD28) receptor sends a costimulatory signal to drive cell activation alongside T cell receptor signaling. Conversely, cytotoxic T lymphocyte-associated protein 4 (CTLA4) opposes T cell activation via competitive, high-affinity interactions for the same ligands as CD28 (ref. ^27^). Pro-inflammatory conventional T (T_conv_) cells selectively express *CTLA4* after activation, whereas anti-inflammatory regulatory T (T_reg_) cells constitutively express *CTLA4* at high levels and further upregulate it upon activation. *ICOS* expression is induced in multiple activated T cell subsets, and its protein product, inducible T cell costimulator (ICOS), binds a unique ligand (ICOSL) to influence T cell polarization and T_reg_ function^28,29^. The association of common genetic variants in this locus with various autoimmune conditions^30,31^ and the clinical efficacy of costimulation-modifying therapies for cancer^32,33^ and autoimmunity^34,35^ together underscore the immunological importance of these genes and motivate deeper understanding of how costimulation is regulated.

The transcriptional programs regulating the CD28 family of costimulatory genes have not been functionally characterized. In recent years, chromatin immunoprecipitation followed by sequencing (ChIP–seq) and ATAC-seq have been widely used to map context-restricted transcription factor binding and CREs^36^, but these methods do not confirm functionality nor do they mechanistically link CREs to their target genes. Consequently, it has been difficult to pinpoint and characterize the causal variant(s) in human 2q33.2 that alter costimulatory gene expression^37–39^ and confer autoimmune disease risk^40–42^. Recently, high-throughput forward genetic screens using CRISPR have been used to functionally link *trans*-regulatory factors and their gene targets^43–51^. Moreover, our group deployed CRISPR activation (CRISPRa) in an immortalized human T cell line to map CREs that regulate immune gene expression^52^. While CRISPRa can systematically identify CREs for which de novo activation is sufficient to alter target gene expression, CRISPRi is uniquely suited to determining the essentiality of CREs for target gene expression in specific cellular contexts^53^. Prior studies have applied this approach in cancer cell lines^54–56^, but technical limitations precluded the application of CRISPRa and CRISPRi at scale in primary human T cells until recently^46^. Using CRISPR-based tools to dissect how *CD28*, *CTLA4* and *ICOS* are dynamically regulated in primary human T cells could uncover insights into molecular mechanisms of immune activation and tolerance. Moreover, functional genomic approaches could simultaneously reveal how regulatory logic has evolved more broadly to tightly orchestrate ancestrally duplicated genes in a complex, multi-gene region.

Here, we report large-scale CRISPRi screens in primary human T_reg_ and T_conv_ cells, tiling single-guide RNA (sgRNA) species across a 1.44-Mb TAD in human chr2q33.2 to discover CREs with gene-, context- and cell type-restricted activity. By assessing how each perturbation affected the expression of each costimulatory gene in each cell population, we overcame the limitations of genomic methods like ChIP–seq and ATAC-seq to functionally link CREs and their gene target(s) in this complex locus. Complementary pooled CRISPR knockout screens identified *trans* regulators of the costimulatory genes, and ATAC-seq profiling of knockout T cells linked *trans* regulators with specific CREs, the chromatin states of which they modify. Our functional genomic studies also uncovered regulatory crosstalk between adjacent genes and a critical role for CTCF in establishing genomic boundaries to coordinate the activity of CREs in the locus. By functionally linking CREs and *trans* regulators, associating them with their gene targets and uncovering how the locus is regulated by CTCF boundary elements, our integrative functional genomics approach systematically decoded the regulatory logic of this complex locus associated with human disease.

## Results

### CRISPRi maps functional CREs in primary human T cell subsets

**Fig. 1:**
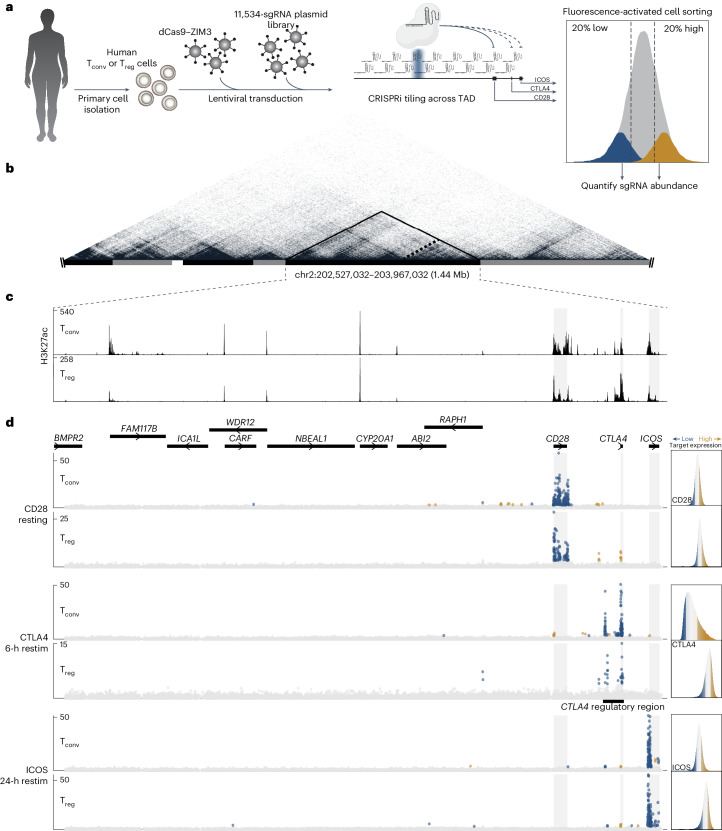
Tiling CRISPRi screens map gene-specific CREs across the costimulatory locus. **a**, Schematic overview of the CRISPRi screening workflow. **b**, Hi-C contact plot from the K562 cells^57^ originally used to design the CRISPRi sgRNA library, with TADs annotated by alternating black and gray bars at the bottom of the figure. The visualized region spans approximately chr2:201,000,000–205,500,000, with the TAD harboring the 2q33.2 costimulatory locus outlined in solid black and expanded in **c**,**d**. The dashed line indicates a subdomain harboring the three target genes of interest. **c**, H3K27ac in T_conv_ cells (top) and T_reg_ cells (bottom) across the TAD designated in **b**. **d**, Gene bodies across the TAD designated in **b** atop CRISPRi tiling screen results for each target gene (rows) in T_conv_ cells (top) and T_reg_ cells (bottom) from two human donors. Each point signifies the genomic position (*x* axis) and −log_10_-transformed unadjusted *P* value (*y* axis) of each sgRNA using the Wald test with Benjamini–Hochberg correction. Blue indicates sgRNA species significantly enriched (adjusted *P* < 0.05) in the lowest 20% of target protein-expressing cells, and gold indicates sgRNA species significantly enriched (adjusted *P* < 0.05) in the highest 20% of target protein-expressing cells. Flow cytometry histograms of target protein expression for each screen are included in the right margin, including the gated populations isolated for sgRNA quantification. The window labeled ‘*CTLA4* regulatory region’ is expanded in Fig. 2. Restim, restimulation.

**Extended Data Fig. 1:**
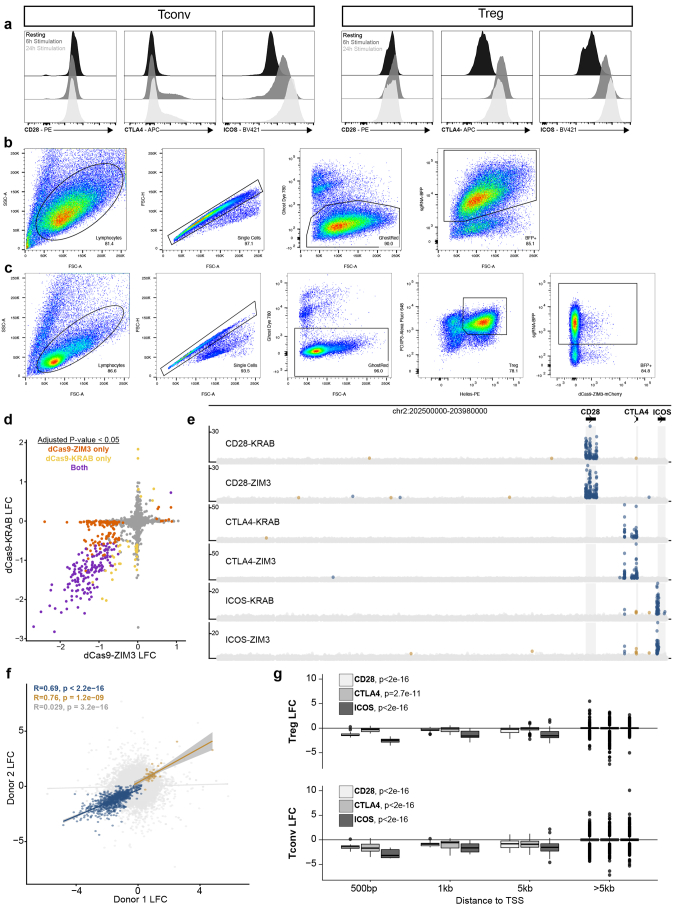
Target protein expression and CRISPRi tiling screen preparations. **a**, Protein expression in T_conv_ (left) and T_reg_ (right) cells for CD28 (sub-left), CTLA4 (sub-middle) and ICOS (sub-right) after 0 hours (black), 6 hours (dark gray), or 24 hours (light gray) restimulation. **b**, Representative FACS gating strategy for CRISPRi screens in T_conv_ cells. **c**, Representative FACS gating strategy for CRISPRi screens in T_reg_ cells. **d**, Examination of high versus low protein bin sgRNA enrichment (log_2_) matched by gene target in bulk CD4^+^ T cells from one human donor with technical replicates. Colors indicate significant (adjusted *P* < 0.05) sgRNA enrichment with the dCas9-ZIM3 (orange), dCas9-KRAB (yellow), or both (purple) CRISPRi systems. **e**, CRISPRi tiling screen results comparing CRISPRi systems (‘ZIM3’, ‘KRAB’) for each target gene (rows) in CD4^+^ cells from one human donor (two technical replicates per condition) across the TAD designated in Fig. 1. CRISPRi tiling results are plotted as in Fig. 1. **f**, Correlation of log_2_(fold change) (LFC) sgRNA enrichment between high (adjusted *P* < 0.05, LFC > 0, gold) and low (adjusted *P* < 0.05, LFC < 0, blue) protein bins matched by cell type and gene target across two biological replicates plotted in Fig. 1d. Gray sgRNAs were not significantly different between low and high bins (adjusted *P* > 0.05). Inset includes Pearson statistics for each group. Lines indicate best fit from a general linear model with 95% confidence interval. **g**, log_2_(fold change) sgRNA enrichment between high versus low protein bins for each gene target and cell type categorized by distance to the target gene transcriptional start site across two biological replicates plotted in Fig. 1d. Significance determined with the one-way ANOVA test for each target per cell type. For all CRISPRi tiling screens, significance was determined using the Wald test with Benjamini-Hochberg correction. Boxplots indicate the sample median (central line), first and third quartiles (box), and 1.5× interquartile range (whiskers).

We set out to discover the CREs regulating *CD28*, *CTLA4* and *ICOS* expression in primary human T cells. Expression of these genes varies between T_conv_ and T_reg_ cell populations as well as under different stimulation conditions for each cell type (Extended Data Fig. 1a). We leveraged recent methodological improvements^46^ to establish a robust CRISPRi-based workflow for mapping CREs in both T_conv_ and T_reg_ cells (Fig. 1a). An annotated TAD in human 2q33.2 harbors all three costimulatory genes (Fig. 1b, black outline)^57^ and contains numerous histone H3 lysine 27 (H3K27) acetylation (H3K27ac) peaks (Fig. 1c), suggestive of active enhancer elements. To map functional CREs regulating the costimulatory genes, we generated an 11,534-sgRNA library tiling across the TAD that could be co-transduced with CRISPRi to compare differential sgRNA abundances in cells with low versus high target protein expression (Fig. 1a and Extended Data Fig. 1b,c). A limited comparison of CRISPRi systems suggested that inactive Cas9 (dCas9) fused to ZIM3^KRAB^ (hereafter referred to as ‘dCas9–ZIM3’) outperforms dCas9 fused to KOX1^KRAB^ (‘dCas9–KRAB’) in primary human CD4^+^ T cells as in other cell types^58,59^, nominating more significant sgRNA species albeit in largely similar genomic regions (Extended Data Fig. 1d,e). Consequently, we deployed the sgRNA library with the dCas9–ZIM3 system for all subsequent experiments.

**Fig. 2:**
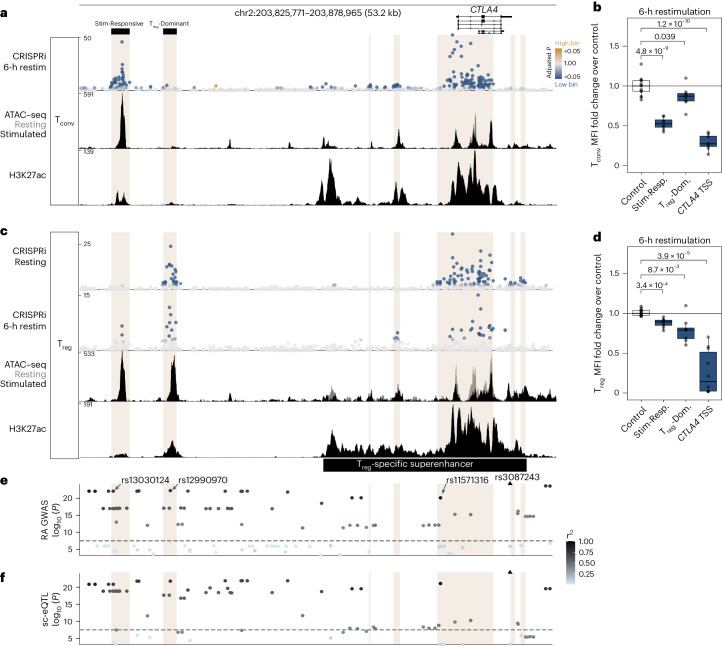
Context-restricted CTLA4 enhancers colocalize with autoimmunity risk variants. **a**, Genomic profiles in the ‘*CTLA4* regulatory region’ annotated in Fig. 1d. Top, CRISPRi tiling in T_conv_ cells restimulated for 6 h. Middle, ATAC-seq profiles of resting (gray) and stimulated (black) T_conv_ cells. Bottom, H3K27ac profile of T_conv_ cells. Beige columns highlight significant CTLA4 CRISPRi regions (Extended Data Fig. 2d,e and the Methods). **b**, Fold change of CTLA4 median fluorescence intensity (MFI) in primary T_conv_ cells (*n* = 2 donors) restimulated for 6 h under arrayed CRISPRi validation of sgRNA species targeting the stimulation-responsive (Stim-Resp.) CiRE (*n* = 4 sgRNAs), the T_reg_-dominant (T_reg_-Dom.) CiRE (*n* = 4) or the *CTLA4* TSS (*n* = 5 for donor 1, *n* = 3 for donor 2) relative to nontargeting controls (*n* = 7 for donor 1, *n* = 6 for donor 2). **c**, Top, CRISPRi tiling results in T_reg_ cells without restimulation. Top middle, CRISPRi tiling results in T_reg_ cells restimulated for 6 h. Bottom middle, ATAC-seq profiles of resting (gray) and stimulated (black) T_reg_ cells. Bottom, H3K27ac profile of T_reg_ cells. The T_reg_-specific H3K27Ac super-enhancer annotation is approximated based on prior studies^41^. **d**, Fold change of CTLA4 median fluorescence intensity in primary T_reg_ cells (*n* = 2 donors) restimulated for 6 h under arrayed CRISPRi validation of sgRNA species targeting the stimulation-responsive CiRE (*n* = 4), the T_reg_-dominant CiRE (*n* = 4) or the *CTLA4* TSS (*n* = 5) relative to nontargeting controls (*n* = 7). **e**, Genetic variants and −log_10_ (*P* value) association with rheumatoid arthritis (RA) risk^30^ colored by linkage disequilibrium to the lead index SNP rs3087243 (triangle). For **e**,**f**, the dashed line indicates *P* = 5 × 10^−8^. GWAS, genome-wide association study. **f**, As in **e** but association with altered *CTLA4* expression in a study of patients with systemic lupus erythematosus and healthy controls^38^. For all CRISPRi tiling screens, each point signifies the genomic position and −log_10_ (unadjusted *P* value) of sgRNA species enriched in the lowest (blue) or highest (gold) 20th percentile expressing cells, with maximum colors signifying adjusted *P* < 0.05 (Wald test with Benjamini–Hochberg correction). sc-eQTL, single-cell expression quantitative trait locus. For **b**,**d**, mean values were compared to that of the control group using two-sided Student’s *t*-test with Holm correction. Box plots indicate the sample median (central line), first and third quartiles (box) and 1.5× interquartile range (whiskers).

**Extended Data Fig. 2:**
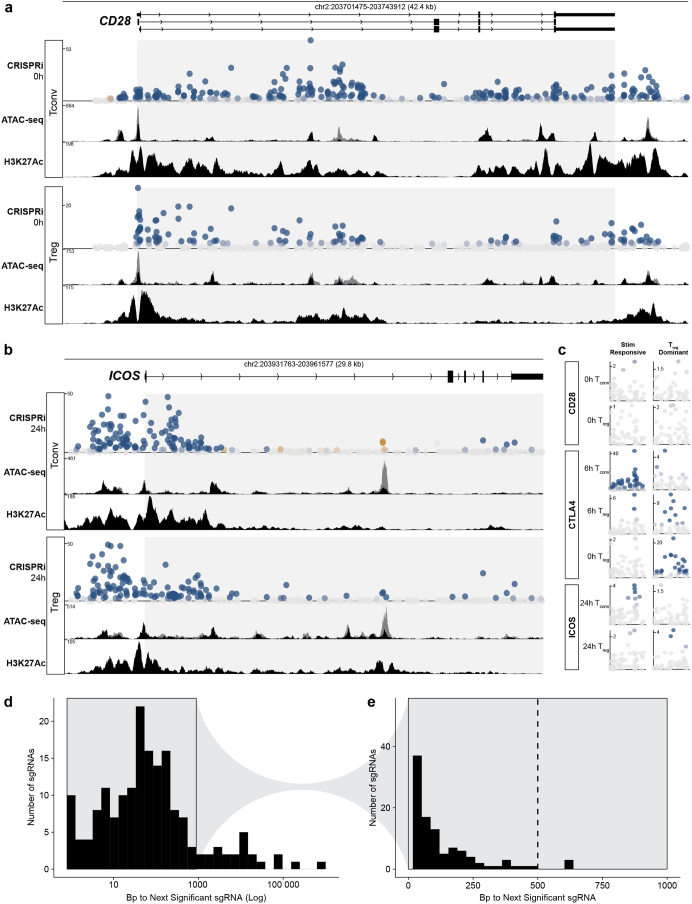
Tiling CRISPRi screens chart *cis* regulation of CD28 and ICOS expression. **a**, Genomic profiles of T_conv_ and T_reg_ cells at the *CD28* gene body as indicated by the isoform diagrams at top. Top: CRISPRi, ATAC-seq, and H3K27ac ChIP-seq profiles in T_conv_ cells. Bottom: CRISPRi, ATAC-seq, and H3K27ac ChIP-seq profiles in T_reg_ cells. For ATAC-seq profiles, accessibility for both resting (gray) and stimulated (black) T_conv_ cells are plotted. Gray region corresponds to the *CD28* gene body. CRISPRi tiling results are plotted as in Fig. 2. **b**, Genomic profiles of T_conv_ and T_reg_ cells at the *ICOS* gene body as indicated by the isoform diagrams at top. Top: CRISPRi, ATAC-seq, and H3K27ac ChIP-seq profiles in T_conv_ cells. Bottom: CRISPRi, ATAC-seq, and H3K27ac ChIP-seq profiles in T_reg_ cells. For ATAC-seq profiles, accessibility for both resting (gray) and stimulated (black) T_conv_ cells are plotted. Gray region corresponds to the *ICOS* gene body. **c**, CRISPR results for each cell type, restimulation condition, and target gene at the Stim-Responsive (chr2:203829159-203831178) and T_reg_-Dominant (chr2:203835010-203836851) CiREs described in Fig. 2. **d**, log-transformed distribution of genomic distance (base pair, bp) for each significant sgRNA (adjusted *P* < 0.05) to the next significant sgRNA across all CTLA4 CRISPRi screens. The subset under 1 kb (gray box with black outline) is re-plotted in **e**. **e**, Non-transformed (linear) representation of the subset of genomic distances plotted in **d** (gray box with black outline) thresholded on inter-sgRNA distances less than 1 kb. A cutoff of 500 bp to the next significant sgRNA (dashed line) at the end of the distribution tail was used to identify CiREs for Fig. 2 (beige regions). For all CRISPRi tiling screens, significance was determined using the Wald test with Benjamini-Hochberg correction.

We performed full CRISPRi tiling screens to identify stimulus-responsive and cell type-specific CREs that control costimulatory gene expression. We isolated donor-matched primary T_conv_ and T_reg_ cells from two human donors, sequentially transduced the cells with lentivirus encoding dCas9–ZIM3 and the sgRNA library and collected samples at the time of peak expression for each gene with or without restimulation (Extended Data Fig. 1a). We identified CRISPRi-responsive elements (CiREs) as candidate CREs influencing target gene expression in each cell type (Fig. 1d). We observed high donor correlation for sgRNA species significantly associated with positive (*R* = 0.76, *P* = 1.2× 10^−9^) and negative (*R* = 0.69, *P* < 2.2× 10^−16^) CRISPRi effects on candidate regulatory elements (Extended Data Fig. 1f). Despite CRISPRi targeting across the entire published TAD, most CiREs were concentrated in individual gene bodies and the *CD28-CTLA4-ICOS* intergenic region exhibiting the most genomic contacts (Fig. 1b, dashed region). CRISPRi signals were generally strongest near each transcriptional start site (TSS) (Extended Data Fig. 1g) and throughout the first introns of target genes, consistent with expected distributions of regulatory elements^60^. We identified additional CiREs both downstream (Extended Data Fig. 2a) and upstream (Fig. 2a–d and Extended Data Fig. 2b) of each gene. These data demonstrate that large-scale CRISPRi tiling screens can be performed in primary human T_conv_ and T_reg_ cell subpopulations to associate noncoding CREs directly with their gene targets.

### CRISPRi associates context-specific CiREs and target genes

Expression of CTLA4 is markedly more variable than expression of CD28 and ICOS; therefore, we sought to identify CiREs responsible for stimulation-dependent CTLA4 upregulation in both T_conv_ and T_reg_ cells as well as those underlying constitutive CTLA4 expression specifically in T_reg_ cells. We examined the annotated ‘*CTLA4* regulatory region’ harboring the majority of CiREs influencing CTLA4 expression (Fig. 1d). Outside of the gene body, expression of CTLA4 in restimulated T_conv_ cells was most sensitive to CRISPRi targeting at a candidate enhancer element ~40 kb upstream of the *CTLA4* TSS (‘stimulation-responsive’), with several other regions exhibiting smaller regulatory effects (Fig. 2a,b). CTLA4 expression in resting T_reg_ cells was largely unresponsive to the stimulation-responsive element but exquisitely sensitive to another candidate enhancer 5 kb downstream (‘T_reg_-dominant’), demonstrating the existence of neighboring enhancer elements that underlie cell- and context-restricted expression of CTLA4 (Fig. 2c). Interestingly, we found that CTLA4 expression in restimulated T_reg_ cells was sensitive to both the T_reg_-dominant CiRE and, to a lesser extent, the stimulation-responsive CiRE (Fig. 2c,d), supporting the idea that distinct *cis* elements can independently or jointly underlie context-specific regulation of target genes. Comparatively, CD28 and ICOS CRISPRi sensitivities varied little between T_conv_ and T_reg_ cells (Extended Data Fig. 2a,b). Of note, we discovered that ICOS expression was subtly sensitive to the stimulation-responsive and T_reg_-dominant CiREs in a cell type-specific manner despite the intervening *CTLA4* gene body (Extended Data Fig. 2c). This finding suggests that presumptively associating candidate CREs with their nearest gene fails to capture the full complexity of *cis* regulation of gene expression^56^. Importantly, although the region upstream of *CTLA4* has been reported as a T_reg_ cell super-enhancer^41^ (Fig. 2c, bottom), we found that much of this region was insensitive to CRISPRi under the conditions assayed. Thus, the context-dependent functional effects we measured throughout this region could not be readily inferred based on ChIP–seq and ATAC-seq alone. We demonstrate that CRISPRi screening uniquely identifies complex, context-restricted CREs that regulate the expression of target genes in specific cell types and activation contexts.

We next explored whether this functional map of CiRE elements could help prioritize human genetic variants conferring risk to T cell-mediated autoimmune conditions like rheumatoid arthritis^61,62^. The biological relevance of CTLA4 regulation is further underscored by clinical efficacy of CTLA4–Ig for rheumatoid arthritis^63^. We annotated CiREs across the *CTLA4* locus by analyzing data from neighboring sgRNA species (Extended Data Fig. 2d,e and Methods). Interestingly, the known *CTLA4* expression quantitative trait locus and index SNP most strongly associated with rheumatoid arthritis risk, rs3087243, resides outside of these CiRE regions (Fig. 2e,f, triangle). By contrast, both rs12990970 in the T_reg_-dominant CiRE and rs13030124 in the stimulation-responsive CiRE are linked to rs3087243 (*r*^2^ = 0.7416 and 0.7316, respectively) and are themselves significantly associated with rheumatoid arthritis risk (Fig. 2e) and *CTLA4* expression (Fig. 2f). Additionally, one variant (rs11571316) in strong linkage disequilibrium with rs3087243 (*r*^2^ = 0.951) is harbored within a CiRE embedded in the *CTLA4* promoter region and the T_reg_ super-enhancer. CRISPRi functional screening can thus help to prioritize candidate causal variants within a haplotype.

### CRISPR knockout with ATAC-seq localizes *trans* effects to CiREs

**Extended Data Fig. 3:**
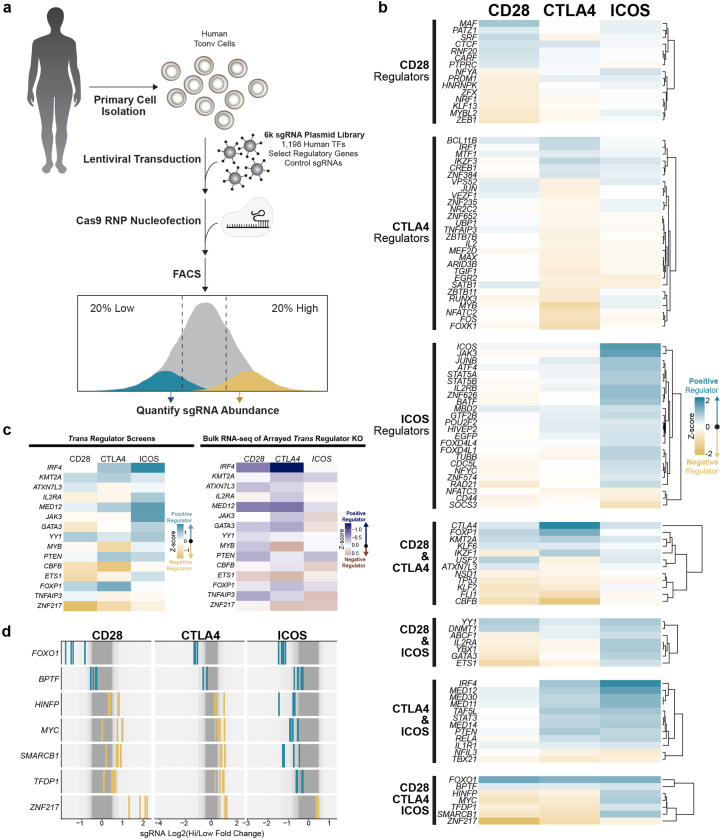
*Trans* regulator screens identify shared and unique gene regulatory modules. **a**, Schematic overview of CRISPR knockout screens to identify *trans* regulators of gene expression in primary human T_conv_ cells from two human donors. **b**, *Trans* regulators grouped by the costimulatory gene product(s) they significantly regulate (adjusted *P* < 0.05) in primary human T_conv_ cells. Positive and negative regulators of gene expression are plotted in blue and gold, respectively. **c**, Effect on costimulatory genes (columns) in the setting of *trans* regulator knockout (rows). Fill colors indicate log_2_(fold change) enrichment from the pooled knockout screen (left, high/low expression) or as measured by RNA-seq in the setting of arrayed *trans* regulator knockout^47^ (right, knockout/control). **d**, log_2_(high/low) enrichment of sgRNAs targeting the set of *trans* regulators significantly regulating all three costimulatory genes. sgRNAs associated with positive or negative *trans* regulators for each costimulatory gene are plotted in blue or gold, respectively. Gray bars indicate the background distribution of all other sgRNAs.

A longstanding challenge has been to identify specific *trans* regulators controlling a given CRE. To more thoroughly characterize the transcriptional regulation of costimulatory genes in T_conv_ cells, we performed CRISPR knockout screens to examine CD28 and ICOS regulation in resting and restimulated primary human T_conv_ cells, respectively (Extended Data Fig. 3a). Integrating these data with published results for CTLA4 (ref. ^47^), we identified factors significantly regulating individual genes, pairs of genes or all three costimulatory genes (Extended Data Fig. 3b). Reassuringly, we noted concordant effects of genes acting in the same biological pathway (for example, Janus kinase 3 (JAK3), signal transducer and activator of transcription 5A (STAT5A) and STAT5B) and regulating stimulation responsiveness (for example, interferon regulatory factor 4 (IRF4) and RelA) (Extended Data Fig. 3b,c). Bulk RNA sequencing of cells with arrayed *trans* regulator knockout largely validated the regulatory effects revealed by the pooled CRISPR screens^47^ (Extended Data Fig. 3c), and replicate sgRNA species targeting the set of *trans* factors significantly regulating all three costimulatory genes exhibited concordant effects (Extended Data Fig. 3d). In sum, these observations confirmed that our systematic CRISPR knockout screens successfully identified *trans* regulators influencing costimulatory gene expression.

**Fig. 3:**
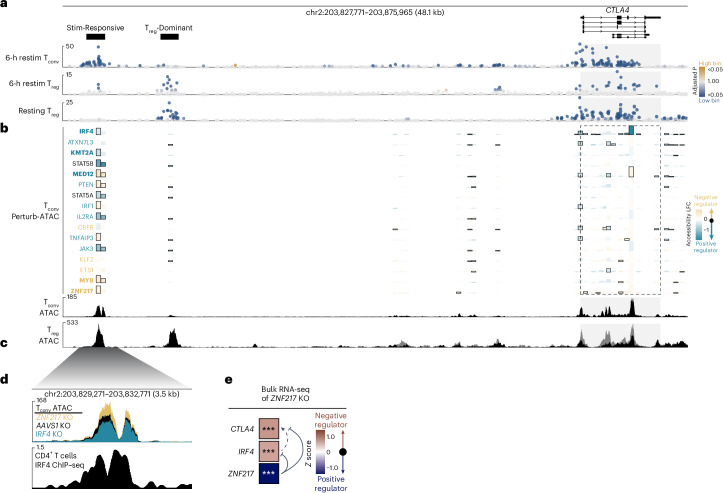
Linking *trans* regulators of CTLA4 to CiREs via ATAC-seq of perturbed cells. **a**, CTLA4 CRISPRi tiling results in T_conv_ cells restimulated for 6 h (top), T_reg_ cells restimulated for 6 h (middle) and resting T_reg_ cells (bottom). CiRE regions are manually annotated. CRISPRi tiling results are plotted as in Fig. 2. **b**, Effect of *trans* regulator knockout on ATAC-seq accessibility^47^ near the *CTLA4* gene body (dashed). Blue indicates positive regulation (that is, *trans* regulator knockout decreases peak accessibility), and gold indicates negative regulation. The height of each peak bar signifies the average of the normalized count values divided by size factors to represent ATAC peak size. Bars outlined in black indicate significant changes in peak accessibility (adjusted *P* < 0.05). Colored *trans* regulator labels indicate those significantly regulating CTLA4 expression either positively (blue) or negatively (gold) according to either the *trans* regulator screens or the arrayed RNA-seq validation, and bolded labels have concordant significant effects between the *trans* regulator screens and the arrayed RNA-seq validation. Bottom, ATAC-seq of *AAVS1*-knockout T_conv_ cell control samples from the profiling experiment. LFC, log_2_-transformed fold change. **c**, Public ATAC-seq of resting (gray) and restimulated (black) T_reg_ cells from a separate experiment. **d**, Top, ATAC-seq of T_conv_ cells with either *ZNF217* (yellow), *AAVS1* control (black) or *IRF4* (blue) knockout (KO). Bottom, public IRF4 ChIP–seq in CD4^+^ T cells. **e**, Changes in *CTLA4* (adjusted *P* = 1.75 × 10^−6^), *IRF4* (adjusted *P* = 2.26 × 10^−7^) and *ZNF217* (adjusted *P* = 2.1 × 10^−16^) expression as measured by bulk RNA-seq in the setting of *ZNF217* knockout in human T_conv_ cells^47^. Significance was determined by two-tailed *t*-test with Benjamini–Hochberg correction in limma. Data supporting positive regulation of IRF4 on *CTLA4* are shown in Extended Data Fig. 3b,c. *P < 0.05, **P < 0.01, ***P < 0.005.

**Extended Data Fig. 4:**
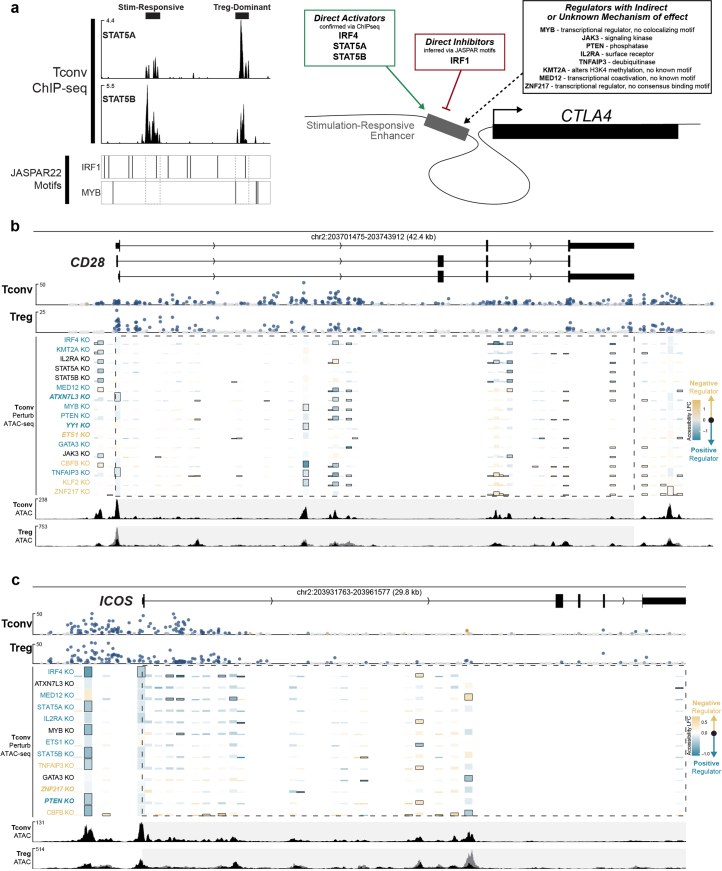
Associating *trans* regulators of *CD28* and *ICOS* with CRISPRi-Responsive Elements via ATAC-seq of perturbed cells. **a**, Evidence of direct *trans* regulator binding at the Stim-Responsive and T_reg_-Dominant CiREs. Left: Publicly available ChIP-seq profiles (top) and motif positions (bottom) for significant *trans* regulators of *CTLA4*. IRF4 ChIP-seq included in Fig. 3d. Right: Schematic of mechanisms of *trans* effects for each significant regulator of *CTLA4*. **b**, CRISPRi tiling, ATAC-seq peak accessibility changes in the setting of *trans* regulator knockout, and reference ATAC-seq tracks for the *CD28* gene body (dashed box). **c**, CRISPRi tiling, ATAC-seq peak accessibility changes in the setting of *trans* regulator knockout, and reference ATAC-seq tracks for the *ICOS* gene body (dashed box). CRISPRi tiling results are plotted as in Fig. 2. For tile plots measuring peak accessibility changes, blue indicates positive regulation (that is, *trans* regulator knockout decreases peak accessibility) and gold indicates negative regulation. The height of each peak bar signifies the average of the normalized count values divided by size factors to signify ATAC-peak size. Bars outlined in black indicate significant changes in peak accessibility (adjusted *P* < 0.05). Colored *trans* regulator labels indicate those significantly regulating costimulatory gene expression either positively (blue) or negatively (gold) according to either the *trans* regulator screens or arrayed RNA-seq validation, and bolded and italicized labels have concordant significant effects between the *trans* regulator screens and arrayed RNA-seq validation. For all CRISPRi tiling screens, significance was determined using the Wald test with Benjamini-Hochberg correction.

**Extended Data Fig. 5:**
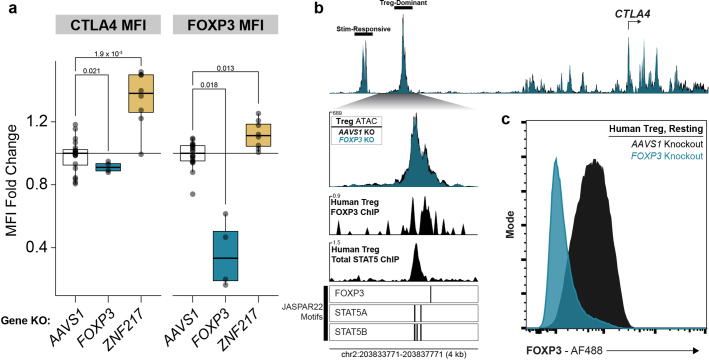
Distinct *cis* and *trans* factors coordinate *CTLA4* expression in T_reg_ cells. **a**, Fold change of CTLA4 (left) and FOXP3 (right) median fluorescence intensity in resting primary T_reg_ cells subjected to arrayed CRISPR knockout of *FOXP3* (*n* = 2 sgRNAs, 4 donors) or *ZNF217* (*n* = 4 sgRNAs for 2 donors, *n* = 2 sgRNAs for 4 donors) relative to *AAVS1* control (*n* = 4 sgRNAs for 2 donors, *n* = 8 sgRNAs for 4 donors). Mean values were compared to the control group using two-sided Student’s *t*-test with Holm correction. Boxplots indicate the sample median (central line), first and third quartiles (box), and 1.5× interquartile range (whiskers). **b**, Normalized ATAC-seq profiles in the *CTLA4* locus of T_reg_ cells subjected to CRISPR-mediated knockout of *AAVS1* (black, control) or *FOXP3* (blue). Tracks represent the consensus normalized read count across T_reg_ cells from two human donors. CiREs are manually annotated. The ATAC-seq peak corresponding to the T_reg_-Dominant CiRE is replotted below with public ChIP-seq data for FOXP3 and total STAT5 in primary human T_reg_ cells and motif positions for FOXP3, STAT5A, and STAT5B. **c**, Representative histograms comparing FOXP3 expression in T_reg_ cells used for ATAC-seq profiling once subjected to *AAVS1* (black, control) or *FOXP3* (blue) knockout.

We sought to link *trans* regulators with the specific CiREs they influence. To this end, we assessed changes in stimulation-responsive CiRE accessibility upon CRISPR-mediated knockout of individual *trans* regulators^47^ (Fig. 3a–c and Extended Data Fig. 4a–c). Next, we used public ChIP–seq datasets, DNA-binding motif localization and annotated gene functions to identify direct effects on the stimulation-responsive CiRE (Extended Data Fig. 4a). Of the candidate regulators, IRF4 is a stimulation-responsive transcription factor that critically regulates T cell function and survival^64^. We found that IRF4 directly bound the stimulation-responsive CiRE and promoted its chromatin accessibility in human CD4^+^ T cells (Fig. 3b,d), consistent with the well-characterized immunological role of the transcription factor and positive effect on CTLA4 expression (Extended Data Fig. 3b,c). Comparatively, the T_reg_ lineage-defining transcription factor^65^ FOXP3 serves as an important positive regulator of CTLA4 (refs. ^66,67^) (Extended Data Fig. 5a) and binds to the T_reg_-dominant CiRE along with STAT5, a transcription factor downstream of interleukin-2 (IL-2) signaling that critically influences T_reg_ cell gene accessibility and expression^68–72^ (Extended Data Fig. 5b). CRISPR knockout in T_reg_ cells revealed that FOXP3 is not required for accessibility of the T_reg_-dominant CiRE despite binding to the element (Extended Data Fig. 5b,c), consistent with reports that FOXP3 can act on chromatin sites made accessible by other factors^73,74^. Together, these findings highlight multiple mechanisms by which validated *trans* factors can act via *cis* elements to influence target gene expression.

**Extended Data Fig. 6:**
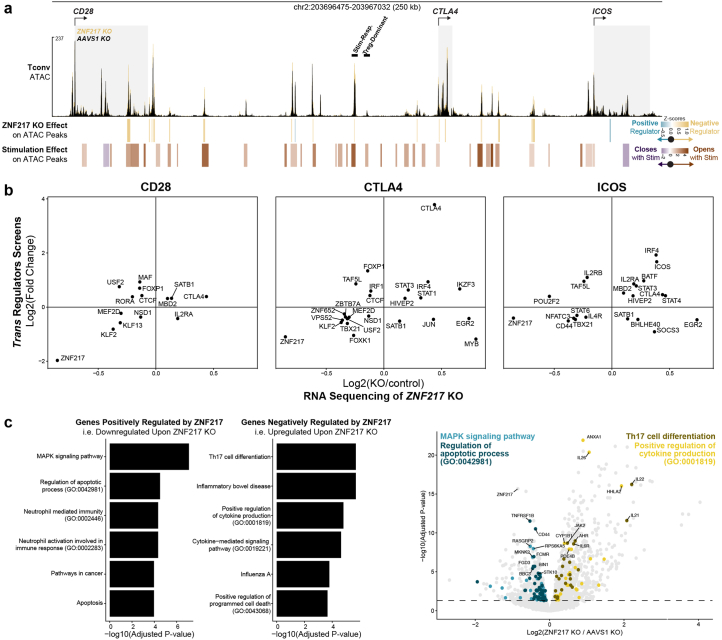
ZNF217 broadly affects gene regulatory networks acting on the costimulatory genes. **a**, Top: ATAC-seq of T_conv_ cells with either *ZNF217* (yellow) or *AAVS1* control (black) knockout across the entire costimulatory gene region tiled by CRISPRi. Costimulatory gene bodies are annotated in gray. Middle: Bars indicate ATAC-seq peaks that significantly (adjusted *P* < 0.05) gain (gold) or lose (blue) accessibility with *ZNF217* knockout using the Wald test with Benjamini-Hochberg correction. Bottom: Bars indicate ATAC-seq peaks that significantly gain (red) or lose (purple) accessibility upon T cell stimulation. **b**, Comparison of the effect of *ZNF217* knockout (FDR < 0.1) on RNA expression of *trans* factors that regulate each costimulatory gene (FDR < 0.1). **c**, Bar plots indicate the top three KEGG and GO terms associated with sets of genes significantly (FDR < 0.05) down- (left) or up-regulated (right) by *ZNF217* knockout. Volcano plot indicates gene expression changes in the setting of *ZNF217* knockout relative to *AAVS1* knockout controls. Down- (blue) or up-regulated (gold) genes associated with the top KEGG or GO term are colored, and the top ten most significantly altered genes associated with any KEGG or GO term are labeled. Dashed line indicates −log_10_(adjusted *P* = 0.05) using the Wald test with Benjamini-Hochberg correction.

We subsequently examined regulators that antagonized costimulatory gene expression. Notably, ZNF217 was the only factor found here to negatively influence all three costimulatory genes in T_conv_ cells (Extended Data Fig. 3b–d), and ATAC-seq profiling revealed that *ZNF217* knockout increased accessibility at the stimulation-responsive CiRE (Fig. 3b,d). Interestingly, we found that ZNF217 negatively regulated CTLA4 in T_reg_ cells (Extended Data Fig. 5a) as it had in T_conv_ cells. ZNF217 has been studied largely in the context of cancer^75^ and is known to associate with various protein complexes to either promote or inhibit target gene expression^76^. Here, we found that *ZNF217* knockout increased accessibility at many other putative CREs in the costimulatory locus in T_conv_ cells (Extended Data Fig. 6a). Furthermore, ZNF217 also influenced the expression of numerous *trans* factors acting on the costimulatory genes (Extended Data Fig. 6b), including *IRF4* in T_conv_ cells (Fig. 3e) and FOXP3 in T_reg_ cells (Extended Data Fig. 5a). These findings reveal cell type-specific regulatory circuits by which ZNF217 inhibits expression of *CTLA4* in T_conv_ and T_reg_ cells at least in part through its effects on IRF4 and FOXP3, respectively. *ZNF217* knockout further resulted in broad transcriptional dysregulation in T_conv_ cells (Extended Data Fig. 6c), adding to the mounting evidence^47,77^ implicating the factor as an important regulator of immune transcriptional programs.

We demonstrate that integrative CRISPR screens and genomic analyses robustly characterize complex gene regulatory relationships influencing costimulatory gene expression by systematically mapping functional noncoding regulatory elements influencing target genes, identifying *trans* regulators influencing those same genes and leveraging functional genomics studies like perturbational ATAC-seq to associate *trans* and *cis* effects.

### Genomic screens revealed regulatory crosstalk between genes

**Fig. 4:**
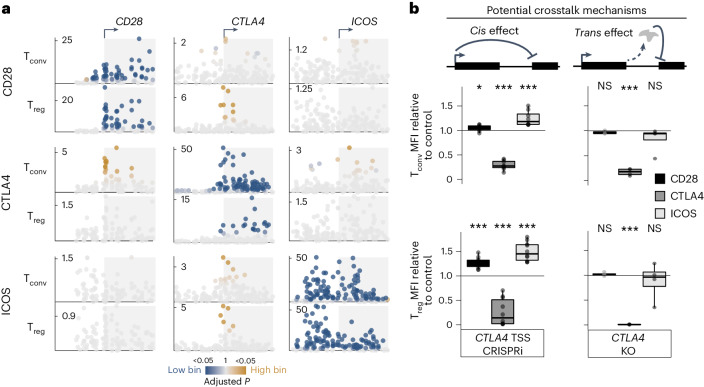
Gene co-regulation evidenced by CRISPRi and *trans* knockout screens. **a**, CRISPRi tiling screen results for each target gene (rows) in T_conv_ cells (top) and T_reg_ cells (bottom) in 10 kb centered on each TSS (columns). CRISPRi tiling results are plotted as in Fig. 2. **b**, Target protein expression in T_conv_ cells (top) and T_reg_ cells (bottom) (*n* = 2 donors) with CRISPRi targeting of the *CTLA4* TSS (left, *n* = 3 sgRNA species for donor 2 T_conv_ samples, *n* = 5 sgRNA species for all other samples) or CRISPR knockout of *CTLA4* downstream of the TSS (right, *n* = 1 sgRNA for the donor 1 T_conv_ CD28 sample, *n* = 2 sgRNA species for all other samples). Plotted are median fluorescence intensity values normalized to those of nontargeting (CRISPRi, *n* = 6 sgRNA species for donor 2 T_conv_ samples, *n* = 7 sgRNA species for all other samples) or *AAVS1*-knockout (*n* = 6 sgRNA species for all samples) controls for each target gene (fill color). Mean values were compared to those of the control group using two-sided Student’s *t*-test with Holm correction (T_conv_ CRISPRi adjusted *P = 0.047, ***P = 1.2 × 10^−10^, ***P = 3.9 × 10^−3^; T_reg_ CRISPRi ***P = 8.2 × 10^−5^, ***P = 4.9 × 10^−5^, ***P = 8.1 × 10^−5^; T_conv_ knockout *P* = 0.73, ***P = 1.8 × 10^−6^, *P* = 0.98; T_reg_ knockout *P* = 1, ***P = 1.2 × 10^−17^, 1). For all panels, *P < 0.05, **P < 0.01, ***P < 0.005; NS, not significant. Box plots indicate the sample median (central line), first and third quartiles (box) and 1.5× interquartile range (whiskers).

**Extended Data Fig. 7:**
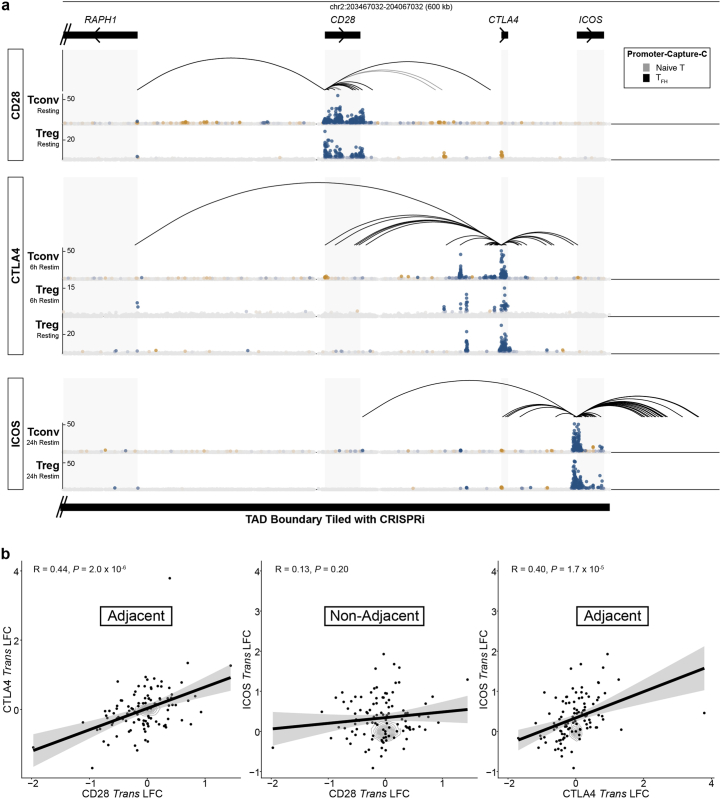
Co-regulation of *trans* factors on adjacent costimulatory genes. **a**, Promoter-Capture-C^78^ for the costimulatory genes in Naive T cells (gray) and T Follicular Helper cells (‘T_FH_’, black) alongside CRISPRi tiling data as plotted in Fig. 2. The figure includes all loops detected for each costimulatory gene. **b**, Correlation of log_2_(fold change) of *trans* regulator effects on all costimulatory receptor pairs. Gray contour plot represents effect sizes of sgRNAs not significantly affecting the expression of any one target gene. Black points indicate *trans* regulators significantly regulating at least one costimulatory gene. Insets include Pearson statistics. Lines indicate best fit from a general linear model with 95% confidence interval.

The sensitivity of all three costimulatory genes to shared *trans* regulators such as ZNF217 led us to explore other mechanisms of coordinated regulation acting in the locus. To our surprise, we observed that CRISPRi-mediated inhibition of each TSS increased expression of the adjacent costimulatory gene(s) (Fig. 4a). That is, CRISPRi-mediated inhibition of the *CTLA4* TSS residing between *CD28* and *ICOS* positively affected the expression of both adjacent genes, whereas targeting *CD28* or *ICOS* only increased expression of the intervening CTLA4. CRISPRi targeting at the *CTLA4* TSS with arrayed sgRNA species validated positive effects on CD28 and ICOS expression relative to that of nontargeting controls (Fig. 4b, left). This effect on neighboring genes was not due to loss of the CTLA4 gene product, as CRISPR-mediated knockout of *CTLA4* had negligible effects on CD28 and ICOS expression (Fig. 4b, right). Importantly, we did not find evidence of promoter homology between adjacent genes, which suggests that these effects are not simply due to promiscuous off-target sgRNA binding to homologous sequences. Instead, we found that promoter-capture-C data^78^ for the costimulatory genes demonstrated extensive interactions between neighboring genes (Extended Data Fig. 7a). Furthermore, we found evidence that adjacent (*CD28-CTLA4* and *CTLA4-ICOS*) but not non-adjacent (*CD28-ICOS*) gene pairs are co-regulated by shared *trans* regulators (Extended Data Fig. 7b). Thus, we discovered that neighboring genes physically interact in *cis*, while shared sets of regulators coordinately influence adjacent costimulatory gene expression in *trans*. In addition to the sharing of stimulation-responsive and T_reg_-dominant CiRE sensitivity by CTLA4 and ICOS (Extended Data Fig. 2c), these findings establish additional regulatory interplay between the costimulatory genes. Moreover, our data reveal an underexplored level of complexity in human gene regulation, providing evidence of complex modes of *cis* and *trans* crosstalk shaping the expression of individual genes in a multi-gene locus.

### CRISPRi-sensitive CTCF sites influence CiRE looping to genes

**Fig. 5:**
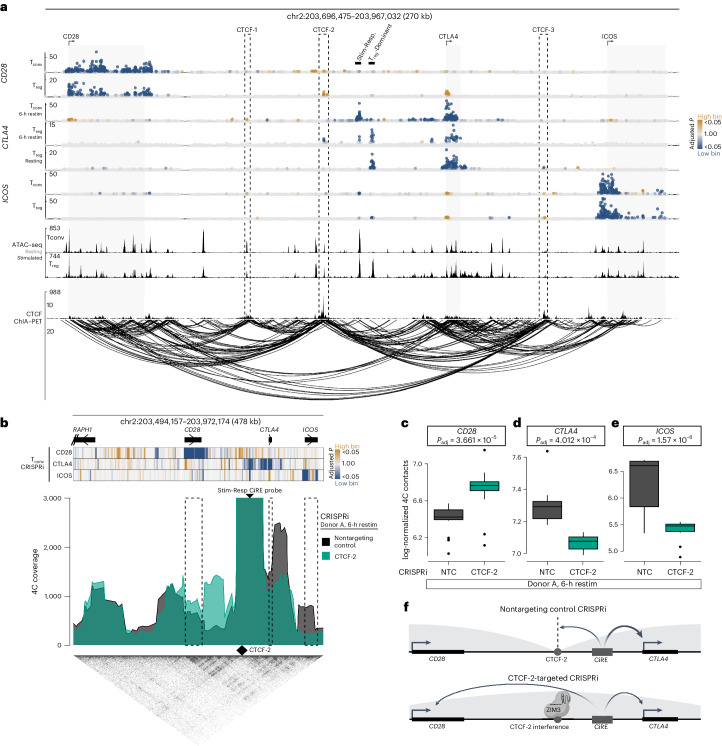
CTCF boundary sites coordinate enhancer looping to costimulatory genes. **a**, Top, CRISPRi tiling screen results for each target gene (rows) in T_conv_ cells (top) and T_reg_ cells (bottom). Gray regions indicate costimulatory gene bodies. Stimulation-responsive and T_reg_-dominant CiREs are manually labeled. CRISPRi tiling results are plotted as in Fig. 2. Middle, ATAC-seq profiles in T_conv_ cells (top) and T_reg_ cells (bottom). Bottom, one-dimensional (1D; top) and two-dimensional (2D; bottom) CTCF-bound genomic contacts identified by ChIA–PET in human CD4^+^ T cells (Methods). Dashed outlines indicate CiREs colocalizing with CTCF ChIA–PET peaks. **b**, A 4C-seq plot anchored on the stimulation-responsive CiRE for T_conv_ cells restimulated for 6 h from one donor (biological replicate of Extended Data Fig. 9d) subjected to CRISPRi-mediated CTCF-2 disruption (green) or a nontargeting control (black). The positions for the stimulation-responsive CiRE probe (serving as the 4C viewpoint) and the CTCF-2 boundary are indicated by arrowheads. Dashed box regions indicate *CD28*, *CTLA4* and *ICOS* gene bodies. CRISPRi tiling screen results in T_conv_ cells are plotted in one dimension (top). Hi-C from CD4^+^ T cells is plotted (bottom). **c**–**e**, Normalized 4C signal intensity comparing nontargeting control (NTC) and CRISPRi-mediated CTCF-2 disruption for the *CD28* (**c**), *CTLA4* (**d**) and *ICOS* (**e**) gene bodies, with results of two-sided *t*-tests with Bonferroni correction. Each point represents the log-transformed 4C-seq signal intensity of each captured genomic region in the respective gene bodies. Results from one donor with accompanying biological replicate data in Extended Data Fig. 9e–g. *P*_adj_, adjusted *P* value. **f**, Schematic overview representing how CTCF-2 disruption alters stimulation-responsive CiRE looping and target gene activation. Box plots indicate the sample median (central line), first and third quartiles (box) and 1.5× interquartile range (whiskers).

Evidence of crosstalk between neighboring genes raised the question of how CREs are ultimately linked specifically to their target genes. Given the role of CTCF in functional three-dimensional (3D) compartmentalization of the linear genome^15^, we next examined CTCF chromatin interaction analysis with paired-end tag sequencing (ChIA–PET)^79^ to understand how the human 2q33.2 locus is structurally organized in primary human CD4^+^ T cells. CTCF ChIA–PET revealed chromatin looping between *CD28-CTLA4* and *CTLA4-ICOS* but not between *CD28-ICOS* promoters and gene bodies (Fig. 5a, bottom), further supporting selective regulatory crosstalk between adjacent genes. This suggests that CTCF binding might establish locus architecture that permits adjacent, but limits non-adjacent, gene interactions.

**Extended Data Fig. 8:**
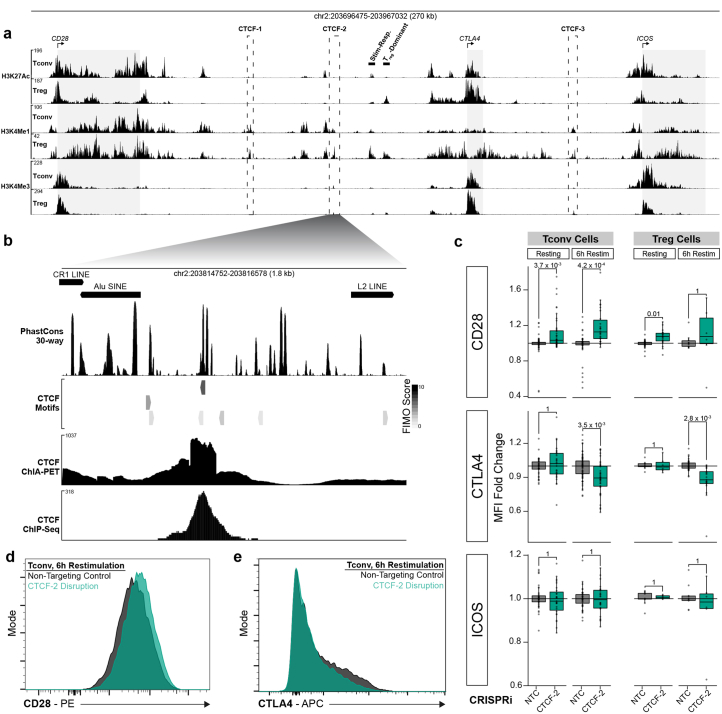
Disruption of CTCF boundary between *CD28* and *CTLA4* alters costimulatory gene expression balance. **a**, Public H3K27ac, H3K4me1, and H3K4me3 ChIP-seq data in primary human T_conv_ (top) and T_reg_ (bottom) cells in the region plotted in Fig. 5a. Gray bars indicate costimulatory gene bodies, and the Stim-Responsive and T_reg_-Dominant CiREs are labeled. Dashed outlines indicate CRISPRi-responsive elements colocalizing with CTCF ChIA-PET peaks as described in Fig. 5. **b**, Zoomed view of the CTCF-2 region indicated in **a**, with track visualizations of retrotransposable elements, PhastCons 30-way conservation, CTCF motif scores and positions, 1-Dimensional CTCF ChIA-PET, and CTCF ChIP-seq. **c**, Median fluorescence intensity (MFI) values after CRISPRi targeting of the CTCF-2 boundary normalized to non-targeting controls (NTC) in T_conv_ (left) and T_reg_ (right) cells at rest (sub-left) or after 6 h restimulation (sub-right). Shown are aggregate data across multiple independent experiments for T_conv_ (NTC: *n* = 39 tests for resting CTLA4 and resting ICOS, 44 for 6 h CD28 and 6 h ICOS, 67 for resting CD28, 72 for 6 h CTLA4; CTCF-2: *n* = 26 for 6 h CD28 and 6 h ICOS, 27 for resting CTLA4 and resting ICOS, 41 for 6 h CTLA4, 42 for resting CD28) and T_reg_ (NTC: *n* = 39 tests for resting CD28 and 6 h CTLA4, 10 for all other conditions; CTCF-2: *n* = 21 tests for resting CD28 and 6 h CTLA4, 6 for all other conditions) cells. Mean values were compared to the respective control group using two-sided Student’s *t*-test with Holm correction. **d**, Representative histograms showing increased CD28 expression in restimulated primary human T_conv_ cells in the setting of CRISPRi-mediated CTCF-2 disruption (green) relative to non-targeting controls (gray). **e**, Representative histograms showing decreased CTLA4 expression in restimulated primary human T_conv_ cells in the setting of CRISPRi-mediated CTCF-2 disruption (green) relative to non-targeting control cells (gray). For all panels: *P < 0.05, **P < 0.01, ***P < 0.005, n.s. not significant. Boxplots indicate the sample median (central line), first and third quartiles (box), and 1.5× interquartile range (whiskers).

We then aligned the ChIA–PET data with our CRISPRi maps to explore how CTCF might influence gene regulation. The peaks of CTCF-mediated 3D genomic contacts colocalized with three CiREs, the perturbation of which subtly yet discordantly altered different costimulatory genes (Fig. 5a, dashed outlines). These CTCF-associated CiREs reside in accessible chromatin marked by histone H3 lysine 4 (H3K4) monomethylation and minimal H3K4 trimethylation but not H3K27ac (Fig. 5a and Extended Data Fig. 8a), consistent with the epigenomic profiles of poised enhancer elements. The largest ChIA–PET peak (labeled ‘CTCF-2’) harbors clustered CTCF binding sites dominated by one highly conserved motif (M1 and M2) (Extended Data Fig. 8b). In large agreement with the tiling screens, arrayed CTCF-2 perturbation with CRISPRi decreased CTLA4 expression relative to that of controls in restimulated T_conv_ and T_reg_ cells but increased CD28 expression in both resting and restimulated T_conv_ cells and resting T_reg_ cells (Extended Data Fig. 8c–e). Together, these findings suggest that a set of CTCF boundaries, most notably CTCF-2, influence the balance of costimulatory gene expression.

**Extended Data Fig. 9:**
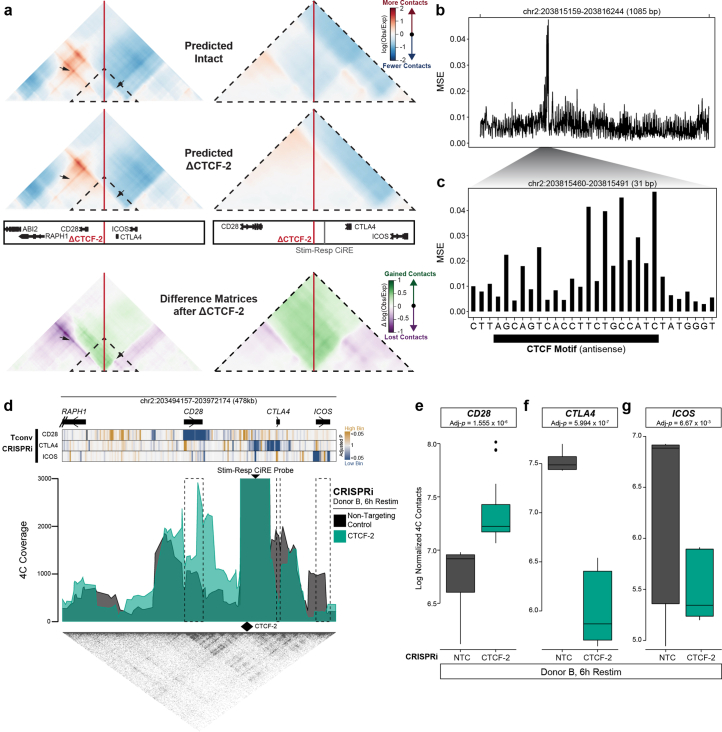
CTCF-2 perturbation alters costimulatory locus conformation and chromatin looping of the Stim-Responsive CiRE. **a**, *In silico* deep learning modeling of genome topology using Akita^81^. Plots span a 1.04-Mb region centered on the CTCF-2 element (red line). Normalized contact frequency matrices for an intact (top left) or computationally-perturbed (‘Predicted ΔCTCF-2’, middle left) CTCF-2 elements are shown, with contacts colored according to higher (red) or lower (blue) contact frequencies than expected. The bottom plots indicate the difference between ‘Predicted Intact’ and ‘Predicted ΔCTCF-2’ plots, with gained (green) and lost (pink) contacts labeled accordingly. A zoomed region (dashed triangle) centered on CTCF-2 is plotted to the right of each *in silico* prediction. **b**, Mean squared error (MSE) of predicted contact matrices with *in silico* 1-bp tiled deletions using Akita. **c**, Subset of **b** demonstrating CTCF motif colocalization with a region of increased topology disruption upon computational perturbation, corresponding to the CTCF-2 region. **d**, 4C-seq plot anchored on the Stim-Responsive CiRE for restimulated T_conv_ cells from one donor (biological replicate of Fig. 5b) subjected to CRISPRi-mediated CTCF-2 disruption (green) or a non-targeting control (‘NTC’, black). The positions for the Stim-Responsive CiRE Probe (serving as the 4C viewpoint) and CTCF-2 boundary are indicated by arrowheads. Dashed box regions indicate *CD28*, *CTLA4*, and *ICOS* gene bodies. CRISPRi tiling screen results in T_conv_ cells are plotted in 1-Dimension (top). **e-g**, Normalized 4C signal intensity comparing non-targeting Control (‘NTC’) and CRISPRi-mediated CTCF-2 disruption for the *CD28* (**e**), *CTLA4* (**f**) and *ICOS* (**g**) gene bodies, with results of two-sided *t* test with Bonferroni correction. Each point represents the log-transformed 4C-seq signal intensity of each captured genomic region in the respective gene bodies. Results from one donor with accompanying biological replicate data in Fig. 5c–e. For all CRISPRi tiling screens, significance was determined using the Wald test with Benjamini-Hochberg correction. Boxplots indicate the sample median (central line), first and third quartiles (box), and 1.5× interquartile range (whiskers).

We then sought to characterize how disrupting the CTCF-2 element altered the balance of *CD28* and *CTLA4* expression. CTCF elements are known to isolate enhancers from nontarget genes^15^; therefore, we hypothesized that CTCF-2 might serve to enforce the relative balance of the opposing CD28 and CTLA4 receptors through conformational control of their shared locus. Deep learning coupled with in silico mutagenesis can robustly model genomic topologies^80,81^; therefore, we used Akita to examine how CTCF-2 perturbation might alter the local 3D conformation of the costimulatory locus. Whereas *CD28* and *CTLA4* regularly reside in separate subdomains defined by a boundary harboring CTCF-2 (‘predicted intact’), disruption of CTCF-2 was computationally modeled to unify the two subdomains (‘predicted ΔCTCF-2’), thereby colocalizing the two genes and their flanking CREs (‘difference matrices after ΔCTCF-2’) (Extended Data Fig. 9a). In silico tiling 1-bp deletions across the boundary region implicated by our CRISPRi screens converged on the CTCF-2 element as most strongly disrupting predicted genomic architecture (Extended Data Fig. 9b,c). These findings suggest that CTCF-2 reinforces the topological conformation of the human costimulatory locus, corroborating a mechanistic model in which disruption of CTCF-2 could alter the *CD28*-*CTLA4* balance by colocalizing the genes and their associated CREs within a single genomic domain.

To experimentally characterize how the CTCF-2 boundary governs 3D chromatin conformation, we performed circular chromosome conformation capture sequencing (4C-seq) in primary T_conv_ cells with or without CRISPRi-mediated perturbation of the CTCF-2 boundary (Fig. 5b–e and Extended Data Fig. 9d–g). Anchoring the 4C-seq assay on the stimulation-responsive CiRE, we found that CTCF-2 boundary perturbation permitted more frequent interactions between the stimulation-responsive CiRE and *CD28* (Fig. 5b,c and Extended Data Fig. 9d,e) at the expense of contacts with *CTLA4* (Fig. 5d and Extended Data Fig. 9f). Interestingly, CTCF-2 perturbation also disrupted interactions with *ICOS* (Fig. 5e and Extended Data Fig. 9g), which shares sensitivity to the stimulation-responsive CiRE with *CTLA4* under full restimulation conditions (Extended Data Fig. 2c) but was not found to be sensitive to CTCF-2 disruption in resting or minimally restimulated cells (Extended Data Fig. 8c). Thus, by mapping the reorganized genomic topology upon CTCF-2 perturbation, we discovered a boundary domain that reinforces stimulation-responsive CiRE prioritization of its primary target gene, *CTLA4*, while simultaneously limiting its effects on expression of a neighboring gene, *CD28* (Fig. 5f).

Altogether, our tiling CRISPRi and *trans* knockout screens revealed a critical regulatory role of CTCF boundary sites in establishing enhancer looping to preferential gene targets. More broadly, our data reaffirm that gene regulation in complex multi-gene loci is susceptible to neighborhood effects and that higher-level genomic organization plays a critical role in restricting enhancer activity to bona fide targets. In summary, we systematically mapped gene-, cell type- and context-specific enhancer elements that regulate costimulatory gene expression under the coordination of *trans* regulators and CTCF boundary elements.

## Discussion

Recent advances in deploying CRISPR technologies in primary human T cells at scale^46^ enabled us to develop an integrative functional genomics approach to discover, validate and functionally disentangle *cis* and *trans* components of complex regulatory circuits key for immune homeostasis. Systematic perturbations of coding and noncoding sequences represent a considerable step beyond genomic profiling approaches such as ChIP–seq and ATAC-seq, which have been instrumental in informing our current understanding of immune cell gene regulatory networks but fail to functionally associate CREs with their gene target(s). Although the CiREs we mapped generally overlapped with chromatin profiles indicative of regulatory elements, only a subset of accessible chromatin regions was sensitive to CRISPRi. As a notable example, most of the well-characterized H3K27ac super-enhancer upstream of *CTLA4* in T_reg_ cells appeared relatively unresponsive to CRISPRi in the specific stimulation contexts we tested. The possibility exists that our assay was not sufficiently sensitive to identify all CREs influencing target gene expression, although we did uncover CRISPRi-responsive sites that did not necessarily exhibit the strongest ATAC-seq or H3K27ac signals. Thus, our functional validation of regulatory element activity reinforces the importance of experimentally annotating noncoding regions^82^. Furthermore, CRISPRi revealed CREs shared by *CTLA4* and *ICOS*, an insight lost with common CRE inference from ChIP–seq and ATAC-seq alone. Future work can use our approach to understand how different CREs operate in specific contexts. The ability to locate critical noncoding sites and functionally connect them directly to target gene(s) in particular cellular contexts moves us beyond traditional chromatin profiling assays and will transform our understanding of how CREs operate in complex genomic loci.

Common genetic variants that influence traits and complex disease risk overwhelmingly localize to noncoding regions of the genome^83^. The noncoding genome remains poorly functionally annotated; therefore, systematic perturbations of disease-associated regions will be crucial to prioritize causal risk variants and functionally link them to gene targets. Furthermore, the lack of conservation of enhancer elements across species^84^ underscores the importance of performing functional experiments directly in human cells as opposed to in model organisms. Numerous publications have associated the human polymorphism rs3087243 with altered *CTLA4* expression^37–39^ and autoimmunity risk^30,31^, but our screens pointed to nearby genetic variants that may individually or cooperatively mediate these expression effects by altering the activity of *CTLA4*-associated CiREs. Importantly, we performed the CRISPRi screens under select stimulation conditions; therefore, the possibility exists that other context-specific enhancer elements could nominate different risk variants under other conditions. Furthermore, our studies were limited to a previously defined TAD^57^, although other studies^78^ have implicated in naive and follicular helper T cells other candidate regulatory regions residing beyond the TAD boundaries tested here (Extended Data Fig. 7a). Thus, additional regulatory elements outside our queried region (and any SNPs within) might still influence costimulatory gene expression. Nonetheless, while we focused primarily on rheumatoid arthritis, our dataset can be used to characterize additional genetic disease associations residing throughout the 2q33.2 locus^85,86^. We not only link new candidate variants to their target genes, but we also discover upstream transcription factors that regulate the chromatin state of the elements that harbor these genetic variants. In sum, we demonstrate the power of functional genomics to decipher gene regulatory networks contributing to disease risk.

The gene products encoded in the *CD28-CTLA4-ICOS* locus tune a delicate costimulatory balance and are under tight regulation. Our perturbation data revealed complex and previously unappreciated circuits controlling expression levels of these receptors, including by ZNF217 acting on *trans* factors with key roles in specific cell types (IRF4 in T_conv_ cells, FOXP3 in T_reg_ cells) that directly occupy context-specific CiREs. Moreover, we demonstrate that systematic genomic studies can characterize how genomic architecture governs CRE and *trans* factor interactions to control expression of specific target genes, especially in complex loci where multiple key target genes neighbor critical enhancer elements. Our CRISPRi tiling data, computational modeling and functional 4C-seq validation showed how CTCF boundaries prioritize enhancer activity to primary gene targets and how boundary disruption permits promiscuous gene regulation. In a locus encoding a critical negative regulatory gene (*CTLA4*) flanked by two activating receptor genes, coordination of enhancer activity to the appropriate target at the appropriate time is of utmost importance: aberrant cellular activation leads to deleterious immune hyper-reactivity, whereas activation blockade impedes immune defense from pathogenic threat. TADs and subTADs function to segregate the genome and organize regulatory processes, but they do so incompletely^87^. Despite intervening CTCF boundary elements of varying strength, our screens identified gene crosstalk in which adjacent costimulatory genes were mutually sensitive to one another. Consistent with previous studies identifying gene crosstalk via competition for shared enhancers^54,88^, we identified stimulation-responsive enhancer sharing between *CTLA4* and *ICOS*, with *CD28* sensitivity to the same element blunted by the CTCF-2 boundary. The neighboring costimulatory genes also could be mutually sensitive due to transcriptional interference^89–95^ or local competition for shared *trans* regulators; therefore, additional experimentation will be needed to explore how these processes might independently or cooperatively affect gene expression balance in multi-gene loci like this.

The *CD28-CTLA4-ICOS* locus is essential for immune regulation and human health. This is evidenced by strong genetic associations with immune dysregulation and the emergence of effective costimulatory modulation treatments for autoimmunity^34,35^ and malignancy^96^. Here, we define coding and noncoding elements that shape the expression of these genes in human T cells. These studies serve as a roadmap for future efforts to define disease-associated functional gene regulatory networks in the relevant primary human cell types. Looking forward, knowledge of specific transcription factors, enhancers and boundary elements that regulate target gene expression in varying immune cell contexts will enable design of complex synthetic circuits to program the expression of immune regulatory products in cellular immunotherapies.

## Methods

### Isolation and culture of human T cells

Peripheral blood mononuclear cell-enriched leukapheresis products (leukopaks) were sourced from Stemcell Technologies. Participants were healthy donors aged 18 years old and older, recruited from the general community and fully consented before donation. Participants were US citizens but otherwise recruited without regard for demographics. CD4^+^CD127^lo^CD25^+^ T_reg_ cells and CD4^+^CD25^−^ T_conv_ cells were isolated using EasySep magnetic selection (Stemcell Technologies, 18063). T_reg_ cell samples were further enriched for purity either before (validation) or after (screen) perturbation as indicated below. All cells were cultured in complete X-VIVO medium (cX-VIVO; Lonza Bioscience, 04-418Q; 5% FCS (R&D Systems, lot M19187), 55 μM 2-mercaptoethanol and 4 mM *N*-acetyl-l-cysteine) unless otherwise specified. T_reg_ and T_conv_ cells were activated by CTS Dynabeads Treg Xpander (Thermo Fisher, 46000D) and CTS Dynabeads CD3/CD28 (Thermo Fisher, 40203D), respectively, with 1:1 cell:bead ratios at 1 × 10^6^ cells per ml in cX-VIVO medium supplemented with recombinant human IL-2 as indicated for each experiment below. Bulk CD4^+^ T cells were isolated separately (Stemcell Technologies, 17952) and otherwise handled the same as T_conv_ cells.

For *trans* regulator screens^47^, primary human CD4^+^CD25^−^ T_conv_ cells isolated as described above were cultured in complete RPMI medium (RPMI (Sigma, R0883), 10% FCS (R&D Systems, lot M19187)), 100 U ml^−1^ penicillin–streptomycin, 2 mM l-glutamine, 10 mM HEPES, 1× MEM non-essential amino acids, 1 mM sodium pyruvate and 50 U ml^−1^ IL-2 (AmerisourceBergen, 10101641)). Cells were activated with ImmunoCult Human CD3/CD28/CD2 T Cell Activator (Stemcell, 10970) at 6.25 μl per 1 × 10^6^ cells.

### Lentivirus production

High-titer lentivirus^46^ was generated using Lenti-X HEK293T cells (Takara Bio, 632180) maintained in complete DMEM (Fisher Scientific, 10566024; 10% FCS (R&D Systems, lot M19187), 100 U ml^−1^ penicillin–streptomycin, 1 mM sodium pyruvate, 1× MEM non-essential amino acids and 10 mM HEPES solution). Lenti-X cells were plated overnight in complete Opti-MEM (Gibco, 31985088; supplemented with 5% FCS (R&D Systems, lot M19187), 1 mM sodium pyruvate and 1× MEM non-essential amino acids) to achieve 85–95% confluency the following morning. Next, Lenti-X cells were transfected with the desired plasmid, the second-generation lentiviral packaging plasmid psPAX2 and the transfer plasmid pMD2.G using Lipofectamine 3000 Transfection Reagent (Fisher Scientific, L3000075). After 6 h, the transfection medium was replaced with complete Opti-MEM supplemented with 1.15× ViralBoost (Alstem Bio, VB100). Lentiviral supernatants were collected 24 and 48 h later, centrifuged at 1,000*g* for 5 min and mixed with one-third volume of Lenti-X Concentrator (Takara Bio, 631232) for 24–96 h at 4 °C. Samples were centrifuged at 1,500*g* for 45 min at 4 °C, resuspended with one-hundredth (screens) or one-tenth (validations) volume cX-VIVO, aliquoted and stored at −80 °C until use. Concentrated lentivirus was titered in a 2× dilution series to identify doses for dCas9–ZIM3 saturation and 50% transduction efficiency of sgRNA libraries.

### Plasmids

The CRISPRi sgRNA library was designed and cloned^52^ to target chr2:202,527,032–203,967,032 (hg38) based on a TAD (extended by 20 kb bilaterally) originally defined in K562 cells^57^. The 11,534-sgRNA library contains every 20-bp protospacer flanked by a 5′-NGG protospacer adjacent motif within the defined region, excluding only sequences (1) containing BstXI or BlpI restriction sites used for cloning and/or (2) perfectly matching additional genomic sites outside the target TAD. Protospacers flanked by adaptor sequences were synthesized by Agilent Technologies, amplified by PCR and cloned into the pCRISPRia-v2 lentiviral vector (Addgene, 84832) using BstXI (NEB, R0113) and Blpl (NEB, R0585)^97^.

The dCas9-ZIM3 plasmid was constructed using NEBuilder HiFi DNA Assembly Master Mix (NEB, E2621), the sequence for the ZIM3^KRAB^–dCas9 domain amplified from pLX303-ZIM3-KRAB-dCas9 (Addgene, 154472) by PCR with primers CM_oligo_1 and CM_oligo_2 (Supplementary Table 1) and the Lenti-SFFV-mCherry-dCas9-VP64 (ref. ^46^) (Addgene, 180263) lentiviral backbone digested with PmeI (NEB, R0560) and BamHI (NEB, R3136).

For arrayed CRISPRi validation experiments, sgRNA sequences were ordered as Ultramers (IDT, 0.2 μM) with flanking adaptors, as in CM_oligo_3, cloned into the pCRISPRia-v2 lentiviral vector (Addgene, 84832) digested with Blpl (NEB, R0585) and BstXI (NEB, R0113) using NEBuilder HiFi DNA Assembly Master Mix (NEB, E2621) and transformed into STBL3 chemically competent cells (QB3 MacroLab). Cultures were grown overnight in the presence of ampicillin (Fisher Scientific, J66972-AC), and plasmid DNA was collected (Zymo Research, D4037, D4203) for lentivirus production. Nontargeting control sgRNA species were sourced from the Dolcetto Human CRISPR Inhibition Pooled Library (Addgene, 1000000114).

### CRISPR interference screens

Primary human T_conv_ cells were activated and maintained with 300 U ml^−1^ rhIL-2. T_reg_ cells were activated with 300 U ml^−1^ rhIL-2 and subsequently maintained with 200 U ml^−1^ rhIL-2. One day after activation, T cells were transduced with saturating doses (1.5–3.5% vol/vol) of 100× concentrated dCas9-ZIM3 lentivirus. The following day, T cells were transduced with sgRNA library virus targeting ~50% transduction efficiency. The following day, cell cultures were split to a density of 1 × 10^6^ cells per ml with fresh cX-VIVO medium supplemented with rhIL-2 and puromycin (final concentration of 2 μg ml^−1^, Fisher Scientific, A1113803). Puromycin selection was confirmed by untransduced T cell death and sgRNA-BFP enrichment as measured by flow cytometry (Thermo Fisher Attune). Cells were split to a density of 1 × 10^6^ cells per ml every 2 d with fresh cX-VIVO medium and rhIL-2. Eight days after activation, one-third of T cells from each donor were restimulated for 24 h with 1 μl per ml Cell Activation Cocktail without brefeldin A (BioLegend, 423302) for subsequent ICOS staining. Eighteen hours later, another one-third of the T cells from each donor were restimulated for 6 h for subsequent CTLA4 staining. At the end of the restimulation period, cells for ICOS (24-h restimulation), CTLA4 (6-h restimulation for both cell types plus 0 h of restimulation for T_reg_ cells only) and CD28 (0 h of restimulation) were pelleted (500*g* for 10 min at 4 °C). Cells were washed with 50 ml cold EasySep buffer (PBS, 2% FCS, 2 mM EDTA (Fisher Scientific, 46-034-CI)), and Dynabeads were removed by magnet. All samples were stained for 30 min at 4 °C with Ghost Dye Red 780 (Tonbo, 13-0865, 1:1,000), and antibodies for ICOS (BioLegend, 313510, 1:25) or CD28 (BioLegend, 302912, 1:25) were included in the appropriate samples. All samples were fixed with the FOXP3 Fix/Perm Buffer Set (BioLegend, 421403) following the manufacturer’s recommended protocol. CTLA4 samples were carried through permeabilization with the FOXP3 Fix/Perm Buffer Set following the manufacturer’s recommended protocol and stained for CTLA4 (BioLegend, 349908, 1:20). For T_reg_ cell screens, all samples were carried through permeabilization and stained with anti-HELIOS (BioLegend, 137216, 1:50) and anti-FOXP3 (BioLegend, 320112, 1:50) antibodies. All samples were stored at 4 °C until flow cytometry.

After fluorescent compensation with single-stained control samples, the highest and lowest 20% expression bins for each target (CD28, CTLA4, ICOS) were sorted into cold EasySep buffer at the Parnassus Flow Cytometry Core Facility or the Gladstone Flow Cytometry Core using BD Aria II, Aria III and Aria Fusion cell sorters. Sorted samples were pelleted and resuspended in 400 μl ChIP lysis buffer (1% SDS, 50 mM Tris, pH 8, 10 mM EDTA) per 5 × 10^6^ cells. Each 400-μl reaction received 16 µl NaCl (5 M) and was incubated at 66 °C overnight. Subsequently, each reaction received 8 µl RNase A (Fisher Scientific, EN0531) and was incubated at 37 °C for 1 h. Next, 8 µl proteinase K (Fisher Scientific, 25530049) was added, and the samples were incubated at 55 °C for 1 h. One phase-lock tube (Quantabio, 2302820) per sample was centrifuged at 20,000*g* for 1 min and received 400 µl phenol–chloroform–isoamyl alcohol (25:24:1). Four hundred microliters of sample was added to each prepared phase-lock tube, which was shaken vigorously and centrifuged at 20,000*g* and 25 °C for 5 min. Aqueous phases were transferred to low-binding tubes (Eppendorf, 022431021) and received 40 µl sodium acetate (Fisher Scientific, 46-033-CI), 1 µl GlycoBlue (Invitrogen, AM9515) and 600 µl isopropanol. Samples were vortexed and frozen at −80 °C for ≥30 min. Frozen samples were centrifuged at 20,000*g* and 4 °C for 30 min, supernatant was removed, and pellets were washed with fresh 70% ethanol and allowed to air dry for 15 min. Genomic DNA pellets were resuspended in Zymo DNA elution buffer (Zymo, D3004-4-10) and reconstituted at 65 °C for 1 h or until dissolution. Sequencing libraries were generated using 3.75 μg genomic DNA per 50-μl PCR reaction with 0.25 μM CM_oligo_4 and 0.25 μM unique p7 reverse primer as in CM_oligo_5 (Supplementary Table 1). PCR reactions were run with the following parameters: 95 °C for 1 min, (95 °C for 30 s, 60 °C for 30 s, 72 °C for 30 s) × 28 cycles, 72 °C for 10 min, hold at 4 °C. Amplicons were purified with DNA Clean & Concentrator-25 kits (Zymo Research, D4033)^98^. One sample (donor 2 T_conv_ cells, ICOS screen) was re-indexed before sequencing. Pooled libraries were sequenced with a custom sequencing primer (CM_oligo_6) on an Illumina NextSeq 500 instrument.

### CRISPR interference screen analysis

Raw Illumina sequencing data were demultiplexed, and fastq files were generated using bcl2fastq (version 2.20.0). Short guide RNA abundance was quantified using MAGeCK (version 0.5.9.4)^99^ with a reference file listing sgRNA sequences, an sgRNA ID and the 5′ genomic position of the sgRNA (hg38). Unnormalized sgRNA count files for each sample were loaded into R (version 4.1.2), and statistical testing of sgRNA effects across donors was performed with DESeq2 (version 1.34.0) using the default Wald test with Benjamini–Hochberg correction^100^. Short guide RNA species with fewer than ten sequencing reads across all samples per condition were excluded from subsequent analyses. To highlight genetic windows of CRISPRi effects for prioritizing variants affecting *CTLA4*, we subsetted our data to all significant sgRNA species identified in any of the *CTLA4* CRISPRi screens (adjusted *P* < 0.05) and examined the distance between adjacent sgRNA species (Extended Data Fig. 2d). Using this strategy, we called CiREs based on runs of sgRNA species less than 500 bp from the previous sgRNA, setting the peak boundaries to the genomic start positions of the first and last sgRNA species within the CiRE.

### CRISPR knockout screens and analysis

CRISPR knockout screens were performed as previously described^47^ to accompany the published CTLA4 data. Cells were isolated and activated as described above. One day after stimulation, cells were transduced with concentrated sgRNA library lentivirus produced as described above. Lentivirus was washed from cells after 24 h in culture. Subsequently, Cas9 ribonucleoproteins (RNPs) were prepared with lyophilized Edit-R crRNA Non-targeting Control #3 (Dharmacon, U-007503-01-05). crRNA species and Edit-R CRISPR–Cas9 Synthetic tracrRNA (Dharmacon, U-002005-20) were resuspended at 160 mM in nuclease-free duplex buffer (IDT, 11-05-01-03), mixed at a 1:1 ratio for an 80 mM solution and incubated at 37 °C for 30 min. Single-stranded donor oligonucleotide enhancer (CM_oligo_7) was added at a 1:1 molar ratio of the final Cas9–guide complex, mixed well by pipetting and incubated for an additional 5 min at 37 °C. Cas9 protein (UCB MacroLab, 40 μM) was added at a 1:1 ratio, mixed thoroughly by pipetting and incubated at 37 °C for 15 min. Prepared Cas9 RNPs were distributed into a 96-well plate. On day 3, stimulated cells were pelleted at 90*g* for 10 min in a centrifuge at 25 °C, the supernatant was removed, and the sample was resuspended at 1 × 10^6^ cells per 20 μl Buffer P3 (Lonza, V4SP-3096). Prepared cells were distributed into the plate with RNPs, mixed gently and transferred to the 96-well Nucleocuvette Plate (Lonza) for nucleofection (Amaxa Nucleofector 96-well Shuttle System). Cells were nucleofected using the pulse code EH-115. Immediately after electroporation, 90 μl complete RPMI prewarmed to 37 °C was added to each well and incubated at 37 °C for 15 min. Cells were pooled, transferred to incubation flasks and diluted with additional medium to a final concentration of 1 × 10^6^ cells per ml. On day 6 after electroporation, cells were fixed, stained and sorted for CD28 (unstimulated) and ICOS (24-h restimulation) staining as described above. sgRNA libraries were generated and sequenced as for the CRISPRi screens. Sequencing data were analyzed in MAGeCK (version 0.5.8) using the ‘count‘ and ‘test‘ commands. All genes with an FDR-adjusted *P* < 0.05 were considered significant.

### Arrayed validation

T_conv_ and T_reg_ cells were magnetically isolated as described above. Immediately after magnetic isolation, CD25^+^CD127^lo^ T_reg_ cells were stained for CD25 (BioLegend, 302618, 1:25), CD127 (Becton Dickinson, 557938, 1:50) and CD4 (BioLegend, 344620, 1:50) in EasySep at 4 °C for 20 min for further purification using fluorescence-activated cell sorting (BD FACSAria Fusion). All samples were activated, sequentially transduced with saturating dCas9-ZIM3 and sgRNA lentiviruses, selected with puromycin and assayed on day 9, as above, with co-staining for CD28 (BioLegend, 302908, 1:25), CTLA4 (BioLegend 349908, 1:20) and ICOS (BioLegend, 313506, 1:25).

For arrayed CRISPR knockout experiments, cells were activated for 2 d before nucleofection. Lyophilized Edit-R crRNA species (Dharmacon) were ordered for each target in an arrayed format. RNPs were prepared, and cells were nucleofected as described above for the CRISPR knockout screens, except using pulse code DS-137. Nucleofected cells were recovered in 80 μl prewarmed cX-VIVO medium at 37 °C for 15 min. Next, nucleofected cells were distributed into 96-well plates and maintained at 1 × 10^6^ cells per ml until analysis. For all validation experiments, protein expression was measured using the Attune NxT Flow Cytometer (Thermo Fisher) and analyzed with FlowJo (version 10.8.1) and R (version 4.1.2). Only samples with 500 or more cells remaining after QC and gating were carried through for analysis. Significance tests were performed with the ggpubr (version 0.4.0) ‘stat_cor‘ and ‘compare_means‘ functions.

### Circular chromosome conformation capture sequencing

T_conv_ cells from two human donors (1 × 10^7^ per donor) were transduced with lentivirus encoding dCas9–ZIM3 and individual CTCF-2 or nontargeting control sgRNA species as described above. Nine days after isolation, cells were restimulated for 6 h and then snap frozen. Cell pellets were thawed, fixed with 1% PFA and repelleted. Cell pellets were resuspended with 500 μl 4C lysis buffer (50 mM Tris-HCl, pH 7.5, 150 mM NaCl, 5 mM EDTA, 0.5% NP-40 (IGEPAL CA-630), 1% Triton X-100 and 1× protease inhibitors (Thermo Fisher, 1862209)). Pellets were pipetted vigorously and lysed on ice for 10 min. Pellets were centrifuged at 750*g* for 5 min at 4 °C and washed twice with cold PBS. Nuclear pellets were resuspended in water and 1× rCutSmart Buffer (NEB, R3104T). SDS (0.25%) and 2.5% Triton X-100 were added for denaturation at 37 °C for 1 h on a thermomixer set to 900 rpm. Genomic DNA was digested with 400–600 UI HindIII-HF (NEB, R3104T) overnight before heat inactivation. Digested genomic DNA was ligated with the T4 DNA ligase system (NEB, M0202T) at 25 °C for 4 h. The mixture was digested with proteinase K (Thermo Fisher, EO0491) and RNase (Roche, 11119915001) and purified by the phenol–chloroform method. DNA pellets were resuspended in TE buffer and subjected to secondary digestion with DpnII overnight (200 UI, NEB, R0543T) before heat inactivation. DNA was again ligated using the T4 DNA ligase system (NEB, M0202T) at 25 °C for 4 h and then pelleted with 60 mM sodium acetate, 3 μg ml^−1^ glycogen and 70% ethanol. Two probe sets spanning the entire stimulation-responsive CiRE were tested, but only the probe covering the latter half of the enhancer region (which aligns with maximum CRISPRi responsiveness) yielded sufficiently diverse libraries and is included here. PCR was performed on 200 ng DNA with CM_oligo_8 and CM_oligo_9 using the Platinum SuperFi DNA Polymerase system (Thermo Fisher, 12351010) with the program (98 °C for 10 s, 52 °C for 10 s, 72 °C for 1 min) × 30 cycles. Final amplified libraries were purified with SPRI cleanup, quantified and sequenced on an Illumina NextSeq 500 instrument.

The 4C sequencing reads were processed and aligned to hg38 using the pipe4C processing pipeline^101^, normalizing to one million reads and using a default window size of 21. The resulting wig files were imported into R and smoothed using spline models (smoothing parameter, 0.75). The 4C method resulted in satisfactory quality parameters according to established guidelines^101^, where over 55% of the reads mapped in the viewpoint chromosome. More than 40% of the total coverage mapped within 1 Mb of the viewpoint, and over 55% of fragments within 100 kb of the viewpoint were captured in any sample. Gene tracks were plotted in Sushi^102^, and smoothed wig files were plotted using R base ‘plot‘. The normalized 4C signal of the captured fragments was extracted for each gene body (chr2:203,706,475–203,738,912, chr2:203,867,771–203,873,965, chr2:203,936,763–203,961,577), log transformed and plotted for viewpoint–gene interactions.

### Akita simulations

The Akita model^81^ was used to predict contact frequency maps around CTCF-2 for both the intact reference sequence and the CTCF-2 deletion. For the intact sequence, a 1,048,576-bp region surrounding CTCF-2 was extracted from hg38. For the single and tiled deletion sequences, the 508-bp CTCF-2 region (chr2:203,815,414–203,815,922) or each base pair in chr2:203,815,159–203,816,244 was removed in silico, respectively, and the sequences were padded on either end to match the intact sequence length. Sequences were inputted into the Akita model, and predictions were generated for human foreskin fibroblast cells. The resulting matrices were compared using MSE. All analyses were performed in Python using Pysam (version 0.15.3), Jupyter (version 1.0.0), Matplotlib (version 3.4.2) and all Akita dependencies.

### Perturb-ATAC-seq

T_reg_ cells from two human donors were isolated and subjected to *FOXP3* or *AAVS1* knockout with CRISPR as described above. Nine days after the initial isolation and stimulation, 15,000 resting T_reg_ cells per sample were resuspended in ATAC lysis buffer (10 mM Tris-HCl, pH 7.4, 10 mM NaCl, 3 mM MgCl_2_, 0.1% IGEPAL), and nuclei were subjected to tagmentation using the Nextera DNA Library Preparation Kit (Illumina). Tagmentation DNA was purified with the MinElute PCR Purification Kit (Qiagen, 28004) and amplified with Phusion High-Fidelity PCR Master Mix (NEB, F531L) using 16 PCR cycles. Amplified libraries were repurified. Fragment distribution of libraries was assessed with the Agilent Bioanalyzer, and libraries were sequenced at low depth on the Illumina NextSeq 500 followed by deep sequencing on the Illumina NovaSeq X using a paired-end 150-bp read configuration. Sequencing was performed at the UCSF Center for Advanced Technology.

ATAC-seq data were analyzed as previously described^47^. In brief, raw sequencing reads were trimmed with cutadapt (version 2.10) to a minimum read length of 20 bp. Reads were aligned to the GRCh38 reference genome using Bowtie 2 (version 2.4.1). Low-quality reads were filtered using SAMtools (version 1.10), reads mapping within ENCODE blacklist regions were removed using bedtools intersect (version 2.29.2), and read duplicates were removed using picard (version 2.23.3). Peaks were called using MACS2 (version 2.2.7.1) before merging biological replicate samples into a consensus peak file. A count matrix was generated by quantifying the number of Tn*5* insertion sites overlapping each consensus peak using the ‘summarizeOverlaps‘ function (GenomicAlignments, version 1.24.0). The ‘estimateSizeFactorsForMatrix‘ function (DESeq2, version 1.34.0) was used to estimate size factors for determination of normalized coverage based on Tn*5* insertion sites. Replicate samples were merged into consensus bigwig files for plotting.

### Genomic data access and processing

Hi-C data^57^ in Fig. 1 were accessed with the 3D Genome Browser^103^. Hi-C data for Fig. 5 were extracted from the ENCODE portal^104^ with the identifier ENCSR421CGL. ATAC-seq profiles were sourced from GSE118189 (ref. ^105^) and GSE171737 (ref. ^47^). ChIP–seq profiles of histone modifications were generated by the NIH Roadmap Epigenomics Mapping Consortium (https://egg2.wustl.edu/). Summary statistics from multi-ancestry GWAS meta-analysis for rheumatoid arthritis^30^ and single-cell genetic analysis of lupus erythematosus^106^ were loaded into R (version 4.1.2), lifted from hg19 to hg38 with a chain file (UCSC) using the ‘liftOver‘ function (rtracklayer version 1.48.0), and linkage disequilibrium relative to the lead variant rs3087243 was calculated with LDlinkR (version 1.2.0). Homology of adjacent gene promoters was examined with Benchling’s alignment tool. Whenever possible, care was taken to select publicly available genomic data gathered from the same primary human T cell subsets under the same activation conditions assayed in the present study. ChIP–seq data for IRF4 (GSM2810038), STAT5A (GSM671400), STAT5B (GSM671402), total STAT5 (GSM1056923) and FOXP3 (GSM1056936) were downloaded from the NIH Sequence Read Archive and aligned to the hg38 reference genome with Bowtie 2 (version 1.17), and coverage tracks were generated with deepTools (version 3.5.2).

*Trans* regulator screening results for *CTLA4*, RNA sequencing in the setting of *trans* regulator knockout and ATAC-seq profiles of *trans* regulator knockout T_conv_ cells are published under GSE171737 (ref. ^47^). To identify transcription factor motifs enriched in ATAC-seq peaks altered by *ZNF217* knockout, bed files of called ATAC-seq peaks gaining or losing accessibility (log_2_ (FC) = |0.3|) were compared to one another using the ‘findMotifsGenome‘ script from HOMER (version 4.11) with ‘–size 350‘. Gene set enrichment of differentially expressed genes between *ZNF217*-knockout and control RNA-seq samples was performed in R (version 4.1.2) with enrichR (version 3.0) using the databases ‘KEGG_2021_Human‘ and ‘GO_Biological_Process_2021‘.

Promoter-capture-C data from E-MTAB-6621 (ref. ^78^) were loaded into R (version 4.1.2) and lifted from hg19 to hg38 with a chain file (UCSC) using the ‘liftOver‘ function (rtracklayer version 1.48.0). CTCF ChIA–PET was generated by the ENCODE Project Consortium^79^ and processed in R (version 4.1.2) to plot only loops (1) detected in samples from both biological replicates within 5 kb and (2) originating and ending in the visualized region. ChIP–seq profiles of CTCF in CD4^+^ T cells from healthy controls were sourced from GSE164215 (ref. ^107^). Genome tracks for gene positions, retrotransposable elements and 30-way PhastCons were downloaded from the UCSC Genome Browser. CTCF motifs were identified with FIMO using the MA0139.1 motif from JASPAR.

Plotting was performed with ggplot2 (version 3.3.5) and pyGenomeTracks (version 3.6).

### Reporting summary

Further information on research design is available in the Nature Portfolio Reporting Summary linked to this article.

## Online content

Any methods, additional references, Nature Portfolio reporting summaries, source data, extended data, supplementary information, acknowledgements, peer review information; details of author contributions and competing interests; and statements of data and code availability are available at 10.1038/s41588-024-01743-5.

### Supplementary information

Reporting Summary

Supplementary Tables Supplementary Tables 1–4.

## Data Availability

Data are available in the Gene Expression Omnbius (GEO) under accession GSE261332.

## Code availability

The code for this paper is available at *Zenodo* (10.5281/zenodo.10858867)^108^.
