# Supplementary material for: Systematic decoding of cis gene regulation defines context-dependent control of the multi-gene costimulatory receptor locus in human T cells
Source: Nat Genet. 2024 May 29;56(6):1156–67. doi: 10.1038/s41588-024-01743-5 (PMC11176074; doi:10.1038/s41588-024-01743-5)
Supplement: Supplementary file 1 — Reporting Summary [file 41588_2024_1743_MOESM1_ESM.pdf]

nature portfolio

Corresponding author(s): Alexander Marson, Chun Jimmie Ye

Last updated by author(s): 03/23/2024

Reporting Summary

Nature Portfolio wishes to improve the reproducibility of the work that we publish. This form provides structure for consistency and transparency in reporting. For further information on Nature Portfolio policies, see our Editorial Policies and the Editorial Policy Checklist.

Statistics

For all statistical analyses, confirm that the following items are present in the figure legend, table legend, main text, or Methods section.

n/a	Confirmed	
☐	☒	The exact sample size (*n*) for each experimental group/condition, given as a discrete number and unit of measurement
☐	☒	A statement on whether measurements were taken from distinct samples or whether the same sample was measured repeatedly
☐	☒	The statistical test(s) used AND whether they are one- or two-sided<br>*Only common tests should be described solely by name; describe more complex techniques in the Methods section.*
☒	☐	A description of all covariates tested
☐	☒	A description of any assumptions or corrections, such as tests of normality and adjustment for multiple comparisons
☐	☒	A full description of the statistical parameters including central tendency (e.g. means) or other basic estimates (e.g. regression coefficient) AND variation (e.g. standard deviation) or associated estimates of uncertainty (e.g. confidence intervals)
☐	☒	For null hypothesis testing, the test statistic (e.g. *F*, *t*, *r*) with confidence intervals, effect sizes, degrees of freedom and *P* value noted<br>*Give P values as exact values whenever suitable.*
☒	☐	For Bayesian analysis, information on the choice of priors and Markov chain Monte Carlo settings
☒	☐	For hierarchical and complex designs, identification of the appropriate level for tests and full reporting of outcomes
☒	☐	Estimates of effect sizes (e.g. Cohen's *d*, Pearson's *r*), indicating how they were calculated

Our web collection on statistics for biologists contains articles on many of the points above.

Software and code

Policy information about availability of computer code

Data collection

CRISPRi Screens
Primary human Tconv cells were activated and maintained in 300U/mL rhIL-2. Treg cells were activated in 300U/mL rhIL-2 and subsequently maintained in 200U/ml rhIL-2. One day after activation, T cells were transduced with saturating doses (1.5-3.5% v/v) of concentrated dCas9-ZIM3 lentivirus. The following day, T cells were transduced with sgRNA library virus targeting ~50% transduction efficiency. The following day, cell cultures were split to 1e6 cells/mL with fresh cX-VIVO supplemented with rhIL-2 and puromycin (2ug/mL final, Fisher Scientific #A1113803). Puromycin selection was confirmed by untransduced T cell death and sgRNA-BFP enrichment as measured by flow cytometry (Thermo Fisher Attune). Cells were split to 1e6 cells/mL every 2 days with fresh cX-VIVO and rhIL-2. Eight days after activation, ⅓ of T cells from each donor were restimulated for 24 hours with 1uL/mL Cell Activation Cocktail without Brefeldin A (Biolegend #423302) for subsequent ICOS staining. Eighteen hours later, another 1/3 of T cells from each donor were restimulated for 6 hours for subsequent CTLA4 staining. At the end of the restimulation period, cells for ICOS (24 hours restimulation), CTLA4 (6 hours restimulation for both cell types plus 0 hours restimulation for Treg cells only), and CD28 (0 hours restimulation) were pelleted (500xG, 10 minutes, 4°C). Cells were washed in 50mL cold EasySep buffer (PBS, 2% FCS, 2mM EDTA (Fisher Scientific #46-034-CI)) and Dynabeads removed by magnet. All samples were stained for 30 minutes at 4°C with Ghost Dye Red 780 (Tonbo #13-0865, 1:1000), and antibodies for ICOS (Biolegend #313510, 1:25) or CD28 (Biolegend #302912, 1:25) were included in the appropriate samples. All samples were fixed with the FOXP3 Fix/Perm Buffer Set (Biolegend #421403) following the manufacturer's recommended protocol. CTLA4 samples were carried through permeabilization with the FOXP3 Fix/Perm Buffer Set following the manufacturer's recommended protocol and stained for CTLA4 (Biolegend #349908, 1:20). For Treg cell screens, all samples were carried through permeabilization and stained with HELIOS (Biolegend #137216, 1:50) and FOXP3 (Biolegend #320112, 1:50) antibodies. All samples were stored at 4°C until FACS.

After fluorescent compensation with single-stained control samples, the highest and lowest 20% expression bins for each target (CD28, CTLA4, ICOS) were sorted into cold EasySep buffer at the Parnassus Flow Cytometry Core Facility (PFCC) and/or Gladstone Flow Cytometry

Core using BD Aria II, Aria III, and Aria Fusion cell sorters. Sorted samples were pelleted and resuspended in 400uL ChIP Lysis Buffer (1% SDS, 50mM Tris, pH 8, 10mM EDTA) per 5e6 cells. Each 400uL reaction received 16µl NaCl (5M) and was incubated at 66°C overnight. Subsequently, each reaction received 8µl RNAse A (Fisher Scientific #EN0531) and was incubated at 37°C 1h. Then, 8µl Proteinase K (Fisher Scientific #25530049) was added and the samples incubated at 55°C 1h. One phase lock tube (Quantabio, #2302820) per sample was spun at 20,000g 1 minute and received 400µl Phenol:Chloroform:Isoamyl Alcohol (25:24:1). 400µl sample was added to each phase lock tube, shaken vigorously, and centrifuged at 20,000g 25°C 5 minutes. Aqueous phases were transferred to low-binding tubes (Eppendorf, #022431021) and received 40µl of Sodium Acetate (Fisher Scientific #46-033-CI), 1µl GlycoBlue (Invitrogen, #AM9515), and 600µl isopropanol. Samples were vortexed and frozen at -80°C ≥30 minutes. Frozen samples were centrifuged 20,000g 4°C 30 minutes, pellets washed with fresh 70% ethanol, and allowed to air dry for 15 minutes. Genomic DNA pellets were resuspended in Zymo DNA elution buffer (Zymo, #D3004-4-10) and reconstituted at 65°C for 1 hour, or until dissolution. Sequencing libraries were generated using 3.75ug genomic DNA per 50uL PCR reaction with 0.25uM CM_oligo_4 and 0.25uM unique p7 reverse primer as in CM_oligo_5 (see Supplementary Information). PCR reactions were run with the following parameters: 95°C 1', [95°C 30", 60°C 30", 72°C 30"] x 28, 72°C 10', 4°C hold. Amplicons were purified with DNA Clean & Concentrator-25 kits (Zymo Research #D4033)99. One sample (Donor 2 Tconv cells, ICOS screen) was re-indexed before sequencing. Pooled libraries were sequenced with a custom sequencing primer CM_oligo_6 on an Illumina NextSeq500 instrument.

CRISPR Knockout Screens
CRISPR knockout screens were performed as previously described47 so as to accompany the published CTLA4 data. Cells were isolated and activated as above. One day after stimulation, cells were transduced with concentrated sgRNA library lentivirus produced as described above. Lentivirus was washed from cells after 24 hours in culture. Subsequently, Cas9 RNPs were prepared with lyophilized Edit-R crRNA nontargeting Control 3 (Dharmacon, #U-007503-01-05). crRNAs and Edit-R CRISPR-Cas9 Synthetic tracrRNA (Dharmacon #U-002005-20) were resuspended to 160mM in nuclease-free duplex buffer (IDT #11-05-01-03), mixed at a 1:1 ratio for a 80 mM solution, and incubated at 37°C 30 minutes. Single-stranded donor oligonucleotides enhancer (ssODN, CM_oligo_7) was added at a 1:1 molar ratio of the final Cas9-Guide complex, mixed well by pipetting, and incubated for an additional 5 minutes at 37°C. Cas9 protein (UCB MacroLab, 40uM) was added at a 1:1 ratio, mixed thoroughly by pipetting, and incubated at 37°C for 15 minutes. Prepared Cas9 ribonucleoproteins (RNPs) were distributed into a 96-well plate. On day 3, stimulated cells were pelleted at 90g for 10 minutes in a 25°C centrifuge, the supernatant removed, and resuspended at 1e6 cells per 20uL Buffer P3 (Lonza #V4SP-3096). Prepared cells were distributed into the plate with RNPs, mixed gently, and transferred to the 96-well Nucleocuvette Plate (Lonza) for nucleofection (DS-137, Amaxa Nucleofector 96-well Shuttle System). Cells were nucleofected using the pulse code EH-115. Immediately after electroporation, 90uL cRPMI prewarmed to 37°C was added to each well and incubated at 37°C 15minutes. Cells were pooled, transferred to incubation flasks, and diluted with additional medium to a final concentration of 1e6 cells/mL. On day 6 after electroporation, cells were fixed, stained, and sorted for CD28 (unstimulated) and ICOS (24 hours restimulation) staining as described above. sgRNA libraries were generated and sequenced as for the CRISPRi screens.

Arrayed Validation
Tconv and Treg cells were magnetically isolated as above. Immediately after magnetic isolation, CD25+CD127low Treg cells were stained for CD25 (Biolegend #302618, 1:25), CD127 (Becton Dickinson #557938, 1:50), and CD4 (Biolegend #344620, 1:50) in EasySep at 4°C 20minutes for further purification using fluorescence-activated cell sorting (BD FACSAria Fusion). All samples were activated, sequentially transduced with saturating dCas9-ZIM3 and sgRNA lentiviruses, puro selected, and assayed on day 9, as above. For arrayed CRISPR KO experiments, cells were activated for 2 days before nucleofection. Lyophilized Edit-R crRNA (Dharmacon) were ordered for each target in an arrayed format. Cells were nucleofected as above except using pulse code DS-137 and recovered in 80uL pre-warmed cXVIVO media. Then, nucleofected cells were distributed into 96-well plates and maintained at 1e6 cells/mL until analysis. For all validation experiments, protein expression was measured using the Attune NxT flow cytometer (Thermo Fisher) and analyzed in FlowJo (v10.8.1) and R (v4.1.2).

4C-Seq
Tconv cells from two human donors (1e7 per donor) were transduced with lentivirus encoding dCas9-ZIM3 and individual CTCF-2 or Non-Targeting Control (NTC) sgRNAs as described above. Nine days after the initial activation, cells were restimulated for 6 hours and then snap frozen. Cell pellets were thawed, fixed with 1% PFA, and re-pelleted. Cell pellets were resuspended with 500uL 4C lysis buffer (50mM Tris-HCl pH 7.5, 150mM NaCl, 5mM EDTA, 0.5% NP-40 (IGEPAL CA-630), 1% Triton-X100, and 1X protease inhibitors (ThermoFisher #1862209)). Pellets were pipetted vigorously and lysed on ice for 10 minutes. Pellets were centrifuged 750g, 5 minutes, 4°C and washed twice with cold PBS. Nuclear pellets were resuspended in H2O and 1X rCutSmart buffer (NEB #R3104T). 0.25% SDS and 2.5% Triton-X100 were added for denaturation at 37°C for one hour on a thermomixer set to 900 rpm. Genomic DNA was digested with 400UI-600UI HindIII-HF (NEB #R3104T) overnight before heat inactivation. Digested genomic DNA was ligated by T4 DNA ligase system (NEB #M0202T) at 25°C 4 hours. The mixture was digested by proteinase K (ThermoFisher #EO0491) and RNase (Roche #11119915001) and purified by phenol-chloroform. DNA pellets were resuspended in TE buffer and subjected to DpnII secondary digestion overnight (200UI, NEB #R0543T) before heat inactivation. DNA was again ligated using the T4 DNA ligase system (NEB #M0202T) at 25°C 4 hours and then pelleted with 60mM sodium acetate, 3ug/ml glycogen, and 70% Ethanol. Two probe sets spanning the entire Stim-Responsive CiRE were tested, but only the probe covering the latter half of the enhancer region (which aligns with maximum CRISPRi responsiveness) yielded sufficiently diverse libraries and is included here. PCR was performed on 200ng DNA with CM_oligo_8 and CM_oligo_9 using Platinum™ SuperFi™ DNA Polymerase system (ThermoFisher #12351010) with the program [98°C 10", 52°C 10", 72°C 1'] x 30 cycles. Final amplified libraries were purified with SPRI clean-up, quantified, and sequenced on an Illumina NextSeq500 instrument.

Perturb-ATAC-Seq
Treg cells from two human donors were isolated and subjected to FOXP3 and AAVS1 knockout with CRISPR as described above. Nine days after the initial isolation and stimulation, 15000 resting Treg cells per sample were resuspended in ATAC Lysis Buffer (10mM Tris-HCl pH7.4, 10mM NaCl, 3mM MgCl2, 0.1% IGEPAL) and nuclei subjected to tagmentation using Nextera DNA Library Preparation Kit (Illumina). Tagmentation DNA was purified with the MinElute PCR Purification Kit (Qiagen #28004) and amplified with Phusion High-Fidelity PCR Master Mix (NEB #F531L) using 16 PCR cycles. Amplified libraries were re-purified. Fragment distribution of libraries was assessed with Agilent Bioanalyzer and libraries sequenced low-depth on Illumina NextSeq 500 followed by deep sequencing on Illumina NovaSeq X using paired-end 150bp read configuration. Sequencing was performed at the UCSF CAT.

Data analysis

Raw sequencing files were processed with bcl2fastq (v2.20.0). Short guide RNA abundances were quantified using MAGeCK (v0.5.9.4) and differential enrichment analyses performed with DESeq2 (v1.34.0) or MAGeCK (v0.5.9.4) as described in Methods. 4C data were analyzed with pipe4c (https://github.com/deLaatLab/pipe4C) and sushi (https://github.com/PhanstielLab/Sushi). Akita modeling (https://github.com/calico/basenji/tree/master/manuscripts/akita) was performed with Pysam (v0.15.3), Jupyter (v1.0.0), and Matplotlib (v3.4.2). ATAC-seq data were processed and analyzed using cutadapt (v2.10), bowtie2 (v2.4.1), SAMtools (v1.10), bedtools intersect (v2.29.2), picard (v2.23.3), MACS2 (v2.2.7.1), GenomicAlignments (v1.24.0), and DESeq2 (v1.34.0). Human genetics and Promoter-Capture-C data were processed with rtracklayer (v1.48.0), and genetics data further processed with LDlinkR (v1.2.0). Public ChIP-seq data were processed with bowtie2 (v1.17) and

deeptools (v3.5.2). Gene enrichment analyses were performed with enrichR (v3.0). R (v4.1.2) was used for all analyses, and plotting was performed with ggplot2 (v3.3.5) and pyGenomeTracks (v3.6). Fluorescence-activated cell sorting was performed using FACSDiva (v8.0.1). Otherwise, flow cytometry was performed using Attune NxT Software (v4.2). All flow cytometry was analyzed and plotted using FlowJo (v10.8.1). The code for this paper is available at https://doi.org/10.5281/zenodo.10858868.

For manuscripts utilizing custom algorithms or software that are central to the research but not yet described in published literature, software must be made available to editors and reviewers. We strongly encourage code deposition in a community repository (e.g. GitHub). See the Nature Portfolio guidelines for submitting code & software for further information.

Data

Policy information about availability of data

All manuscripts must include a data availability statement. This statement should provide the following information, where applicable:

- Accession codes, unique identifiers, or web links for publicly available datasets
- A description of any restrictions on data availability
- For clinical datasets or third party data, please ensure that the statement adheres to our policy

HiC data in Figure 1 were accessed with the Yue Lab's 3D Genome Browser (Northwestern University). HiC data for Figure 5 were extracted from the ENCODE portal with the identifier ENCSR421CGL. ATAC-Seq profiles were sourced from GSE118189105 and GSE17173747. ChIP-Seq profiles of histone modifications were generated by the NIH Roadmap Epigenomics Mapping Consortium (https://egg2.wustl.edu/). Summary statistics from trans-ethnic GWAS meta-analysis for rheumatoid arthritis (ref 30) and single cell genetic analysis of lupus erythematosus (ref 106) were sourced from the respective publications. ChIP-Seq data for IRF4 (GSM2810038), STAT5A (GSM671400), STAT5B (GSM671402), Total STAT5 (GSM1056923), and FOXP3 (GSM1056936) were downloaded from the NIH Sequence Read Archive. Trans regulator screening results for CTLA4, RNA sequencing in the setting of trans regulator knockout, and ATAC-seq profiles of trans regulator knockout Tconv cells are published under GSE17173747. Promoter-Capture-C data was sourced from E-MTAB-662178. CTCF ChIA-PET was generated by the ENCODE Project Consortium. ChIP-seq profiles of CTCF in CD4+ T cells from healthy control subjects were sourced from GSE164215107. Genome tracks for gene positions, retrotransposable elements, and 30-way PhastCons were downloaded from the UCSC Genome Browser. CTCF motifs were identified with FIMO using the MA0139.1 motif from JASPAR2022 (https://jaspar2018.genereg.net/matrix/MA0139.1/). Data generated from this publication are available in GEO under accession GSE261332.

Research involving human participants, their data, or biological material

Policy information about studies with human participants or human data. See also policy information about sex, gender (identity/presentation), and sexual orientation and race, ethnicity and racism.

Reporting on sex and gender	N/A
Reporting on race, ethnicity, or other socially relevant groupings	N/A
Population characteristics	N/A
Recruitment	Recruitment was conducted by Stemcell Technologies.
Ethics oversight	Stemcell Technologies provided Human Peripheral Blood Leukopaks using Institutional Review Board (IRB)-approved consent forms and protocols

Note that full information on the approval of the study protocol must also be provided in the manuscript.

Field-specific reporting

Please select the one below that is the best fit for your research. If you are not sure, read the appropriate sections before making your selection.

☒ Life sciences ☐ Behavioural & social sciences ☐ Ecological, evolutionary & environmental sciences

For a reference copy of the document with all sections, see nature.com/documents/nr-reporting-summary-flat.pdf

Life sciences study design

All studies must disclose on these points even when the disclosure is negative.

Sample size	CRISPRi tiling screens, knockout screens, ATAC-seq, and 4C-seq were performed in primary human T cells from 2 independent donors to identify phenotypes reproducible across distinct biological replicates. All subsequent validation experiments were conducted in cells from 2 or more independent donors as indicated in figure legends. By comparing across independent donors, we sought to identify and validate reproducible phenotypes.
Data exclusions	Flow cytometry samples from arrayed validation experiments with less than 500 cells were excluded from subsequent analyses. For CRISPRi tiling screens, sgRNAs with fewer than 10 sequencing reads across all samples for each condition were excluded from subsequent analyses.
Replication	All experiments were performed in multiple technical (ZIM3 vs KRAB comparison only) and/or biological replicates (all other experiments). Only the significant findings reproducible across replicates were followed up on for subsequent study, including all major CRISPRi, KO, 4C-seq,

	and ATAC-seq findings emphasized in the paper. In some circumstances, multiple replicate experiments had already been performed for other purposes, so the sample size varies condition to condition. For instance, in Supplemental Figure 8, more experiments had been performed examining CD28 in Resting cells and CTLA4 in 6h Restimulated cells given that those stimulation conditions match the conditions for CD28 (resting) and CTLA4 (6h restimulation) in the CRISPRi tiling screens. All CTCF-2 validation data generated over the life of the study were included in the figure unless subjected to exclusion per the criteria specified above.
Randomization	Primary human T cells were isolated from peripheral blood leukopaks provided by Stemcell Technologies isolated from human donors >18 years old without regard for demographics. All comparison conditions (e.g. control vs KO) were performed in donor-matched cells and thus internally controlled.
Blinding	All samples were handled equally and unblinded given that data from multiple biological and/or technical replicates were pooled for analysis.

Reporting for specific materials, systems and methods

We require information from authors about some types of materials, experimental systems and methods used in many studies. Here, indicate whether each material, system or method listed is relevant to your study. If you are not sure if a list item applies to your research, read the appropriate section before selecting a response.

Materials & experimental systems

n/a	Involved in the study
☐	☒ Antibodies
☐	☒ Eukaryotic cell lines
☒	☐ Palaeontology and archaeology
☒	☐ Animals and other organisms
☒	☐ Clinical data
☒	☐ Dual use research of concern
☒	☐ Plants

Methods

n/a	Involved in the study
☒	☐ ChIP-seq
☐	☒ Flow cytometry
☒	☐ MRI-based neuroimaging

Antibodies

Antibodies used	ICOS (Biolegend #313510, Biolegend #313506, Biolegend #313524), CD28 (Biolegend #302912, Biolegend #302908), CTLA4 (Biolegend #349908), HELIOS (Biolegend #137216), FOXP3 (Biolegend #320112), CD25 (Biolegend #302618), CD127 (Becton Dickinson #557938), CD4 (Biolegend #344620)
Validation	Antibodies for CD28, CTLA4, and ICOS were first tested in the setting of CD28, CTLA4, or ICOS gene knockout/knockdown cells, with significant differences in protein signal noted between control and perturbed cells. When staining for Helios, Foxp3, CD25, and CD127 to further purify Treg cells, Treg samples were always stained alongside Tconv cell samples to inform correct gating, with significant differences in FOXP3/Helios and CD25/CD127 signals noted between Treg and Tconv cell samples. CD4 staining was first validated by comparing unpurified Leukopak cells with magnetically-isolated Tconv and/or Treg cells. The detected signals for CD28 (https://www.biolegend.com/en-gb/products/apc-anti-human-cd28-antibody-626?GroupID=BLG5919, https://www.biolegend.com/en-ie/products/pe-anti-human-cd28-antibody-630), CTLA4 after PMA/Ionomycin stimulation (https://www.biolegend.com/nl-nl/products/apc-anti-human-cd152-ctla-4-antibody-6999?GroupID=BLG9072), ICOS after PHA stimulation (https://www.biolegend.com/fr-ch/products/apc-anti-human-mouse-rat-cd278-icos-antibody-2566?GroupID=BLG3831, https://www.biolegend.com/en-ie/products/fitc-anti-human-mouse-rat-cd278-icos-antibody-2481, https://www.biolegend.com/en-ie/products/brilliant-violet-421-anti-human-mouse-rat-cd278-icos-antibody-8876), Helios (https://www.biolegend.com/fr-ch/products/pe-anti-mouse-human-helios-antibody-6481), FOXP3 (https://www.biolegend.com/fr-ch/products/alexa-fluor-488-anti-human-foxp3-antibody-2914), CD25 after PHA stimulation (https://www.biolegend.com/fr-ch/products/alexa-fluor-647-anti-human-cd25-antibody-3254), CD127 (https://www.bdbiosciences.com/en-us/products/reagents/flow-cytometry-reagents/research-reagents/single-color-antibodies-ruo/pe-mouse-anti-human-cd127.557938), and CD4 (https://www.biolegend.com/fr-ch/products/pacific-blue-anti-human-cd4-antibody-6507) were compared to the published validation data on manufacturers' websites.

Eukaryotic cell lines

Policy information about cell lines and Sex and Gender in Research

Cell line source(s)	Lenti-X™ 293T Cell Line (Takara #632180)
Authentication	The cell line was not re-authenticated after purchasing from Takara.
Mycoplasma contamination	The cell line was not re-tested for Mycoplasma after purchasing from Takara.
Commonly misidentified lines (See ICLAC register)	N/A

Plants

Seed stocks

Report on the source of all seed stocks or other plant material used. If applicable, state the seed stock centre and catalogue number. If plant specimens were collected from the field, describe the collection location, date and sampling procedures.

Novel plant genotypes

Describe the methods by which all novel plant genotypes were produced. This includes those generated by transgenic approaches, gene editing, chemical/radiation-based mutagenesis and hybridization. For transgenic lines, describe the transformation method, the number of independent lines analyzed and the generation upon which experiments were performed. For gene-edited lines, describe the editor used, the endogenous sequence targeted for editing, the targeting guide RNA sequence (if applicable) and how the editor was applied.

Authentication

Describe any authentication procedures for each seed stock used or novel genotype generated. Describe any experiments used to assess the effect of a mutation and, where applicable, how potential secondary effects (e.g. second site T-DNA insertions, mosiacism, off-target gene editing) were examined.

Flow Cytometry

Plots

Confirm that:

☐ The axis labels state the marker and fluorochrome used (e.g. CD4-FITC).

☐ The axis scales are clearly visible. Include numbers along axes only for bottom left plot of group (a 'group' is an analysis of identical markers).

☐ All plots are contour plots with outliers or pseudocolor plots.

☐ A numerical value for number of cells or percentage (with statistics) is provided.

Methodology

Sample preparation

CRISPRi Screens
Eight days after activation, a fraction of T cells from each donor were restimulated for 24 hours with 1uL/mL Cell Activation Cocktail without Brefeldin A (Biolegend #423302) for ICOS staining. Eighteen hours later, a fraction of T cells from each donor were restimulated for 6 hours with 1ul/mL Cell Activation Cocktail without Brefeldin A for CTLA4 staining. At the end of the restimulation period, samples were harvested for fluorescence-activated cell sorting (FACS). Cells for ICOS (24 hours restimulation), CTLA4 (6 hours restimulation for both cell types and 0 hours restimulation for Treg cells only), and CD28 (0 hours restimulation) were spun down at 500xG, 10 minutes, 4°C. After spinning, cells were washed in 50mL cold EasySep buffer (PBS, 2% FCS, 2mM EDTA), applied to a magnet for removing Dynabeads, and transferred to a new tube. All samples were stained for 30 minutes at 4°C with Ghost Dye Red 780 (Tonbo #13-0865), and antibodies for ICOS (Biolegend #313510) and CD28 (Biolegend #302912) were included in the appropriate samples. After surface staining, cells were washed twice in cold EasySep buffer and fixed with the FOXP3 Fix/Perm Buffer Set (Biolegend #421403) at room temperature for 30 minutes. After fixation, cells were spun at 750xG, 10 minutes, 4°C. ICOS and CD28 samples were resuspended at 40e6 cells/mL in cold EasySep buffer and stored at 4°C until FACS. Samples for Total CTLA4 staining were washed and permeabilized in FOXP3 Perm/Wash Buffer (Biolegend #421403) at room temperature for 15 minutes, spun, and stained with anti-CTLA4 antibody (Biolegend #349908) in 1x FOXP3 Perm/Wash Buffer at room temperature for 30 minutes. Cells were washed twice in cold EasySep buffer and resuspended at 40e6 cells/mL in cold EasySep buffer and stored at 4°C until FACS. For Treg cell screens, all samples were carried through permeabilization and stained with HELIOS (Biolegend #137216) and FOXP3 (Biolegend #320112) antibodies.

After fluorescent compensation with single-stained control samples, the highest and lowest 20% expression bins for each target (CD28, CTLA4, ICOS) were sorted into cold EasySep buffer at the Parnassus Flow Cytometry Core Facility (PFCC) and/or Gladstone Flow Cytometry Core using Aria II, Aria III, and Aria Fusion (BD Biosciences) cell sorters and FACSDiva software (v8.0.1).

Arrayed Validation
Tconv and Treg cells were magnetically isolated as above. Immediately after magnetic isolation, CD25+CD127low Treg cells were stained for CD25 (Biolegend #302618, 1:25), CD127 (Becton Dickinson #557938, 1:50), and CD4 (Biolegend #344620, 1:50) in EasySep at 4°C 20minutes for further purification using fluorescence-activated cell sorting (BD FACSAria Fusion). All samples were activated, sequentially transduced with saturating dCas9-ZIM3 and sgRNA lentiviruses, puro selected, and assayed on day 9, as above, with co-staining for CD28 (Biolegend #302908, 1:25), CTLA4 (Biolegend #349908, 1:20), and ICOS (Biolegend #313506, 1:25). For all validation experiments, protein expression was measured using the Attune NxT flow cytometer (Thermo Fisher) and analyzed in FlowJo (v10.8.1) and R (v4.1.2).

Instrument

BD Aria II, Aria III, and Aria Fusion cell sorters; Thermo Fisher Attune NxT flow cytometer

Software

FACSDiva v8.0.1; Attune NxT Software v4.2; FlowJo v10.8.1; R v4.1.2

Cell population abundance

For CRISPRi screens, the top and bottom 20% of cells were sorted for subsequent molecular analysis of sgRNA enrichment. For arrayed validation experiments, the Cell Count and Median Fluorescence Intensity value for each target of interest was exported from FlowJo for each sample analyzed. Summary plots were generated in R.

Gating strategy

Viable cells were gated with FSC-A and SSC-A, singlets gated with FSC-A and FSC-H, and GhostRed viability dye-negative cells

Gating strategy

were selected. For Treg sorts, Foxp3-positive Helios-positive gates (CRISPRi screens) and CD25-High CD127-Low gates (arrayed validations) were set according to donor-matched Tconv samples. Treg gates were included, where applicable, and then all samples were gated on BFP positivity for sgRNA transduction based on untransduced control cells.

☒ Tick this box to confirm that a figure exemplifying the gating strategy is provided in the Supplementary Information.
